# A target-domain augmentation and class-wise alignment framework for few-shot cross-condition bearing fault diagnosis

**DOI:** 10.1371/journal.pone.0361486

**Published:** 2026-10-29

**Authors:** Yijia Hu, Ying Gu, Yanjie Su, Chuan Zhao

**Affiliations:** 1 Industrial Technology Center, North China Institute of Aerospace Engineering, Langfang, China; 2 Hebei Province Technology Innovation Center for Aerospace Composite Structural Design and Manufacturing, North China Institute of Aerospace Engineering, Langfang, China; 3 School of Mechanical and Electrical Engineering, North China Institute of Aerospace Engineering, Langfang, China; Zhejiang University, CHINA

## Abstract

Cross-condition bearing fault diagnosis often has to adapt to a new operating condition with only a few labelled target samples. This setting creates two coupled problems. First, the sparse target support set cannot describe the target fault distribution sufficiently. Second, the same fault class may occupy different feature regions across source and target conditions, which weakens source-domain pretrained classifiers. Existing methods usually improve few-shot learning or reduce domain shift, but they rarely enrich the labelled target-class representation before class-wise source-target alignment. To address this limitation, this paper proposes a target-domain augmentation and class-wise alignment framework for few-shot cross-condition bearing fault diagnosis. The rime optimization algorithm (RIME) is first used to optimize the variational mode decomposition (VMD) parameters *K* and α, and fault-sensitive components are selected using envelope entropy and adjacent modal-frequency overlap. The reconstructed signal is converted into symmetric dot pattern (SDP) images to provide a noise-suppressed polar representation. The fast generative adversarial network (FastGAN) then generates auxiliary target-domain SDP samples from the limited labelled target support set, making each target fault class less sparse. On this basis, class-wise maximum mean discrepancy (CMMD) is embedded into a Swin Transformer classifier to align source and target features within each known fault category. Experiments on the Case Western Reserve University (CWRU) and Xi’an Jiaotong University–Shenyang Blower Works (XJTU-SY) datasets show that the proposed framework improves target-domain few-shot adaptation, achieving 93.33% and 97.58% accuracy under the G20 and G50 XJTU-SY target-augmentation settings, respectively.

## Introduction

Rolling bearings are used in motors, gearboxes, and other rotating machines. Their condition affects the safety and continuity of industrial production. Data-driven fault diagnosis has improved bearing monitoring by learning discriminative features from vibration signals [1–3]. In practice, however, two assumptions are often difficult to satisfy. The first is that enough labelled fault samples are available. The second is that the training and testing data follow similar distributions. Fault samples are hard to collect because machines usually operate in a healthy state, while severe faults often lead directly to maintenance. Changes in speed, load, transmission path, sensor position, and noise also change the measured vibration response. A model trained under one condition may therefore perform poorly under another condition with only a few labelled samples.

Several research lines have addressed limited labelled data in fault diagnosis. Transfer learning reuses feature extractors trained on source-domain vibration data, and supervised joint distribution adaptation has been used for cross-condition bearing diagnosis [4]. This strategy reduces the need for target labels, but its effect depends on the relevance of the source domain. When the target condition differs strongly from the source condition, fine-tuning with only a few target samples may still be unstable. Self-supervised and contrastive learning methods approach the same problem through representation learning. They learn reusable features from unlabelled or weakly labelled signals through reconstruction, prediction, or contrastive objectives [5,6]. For example, cross-sensor self-contrastive learning (CSSCL) has been used for nondestructive motor fault detection under label-scarce conditions [7]. These methods improve feature initialization and robustness, but they do not increase the number of labelled target samples.

Few-shot learning makes better use of small support sets. Metric-based methods classify query samples by comparing them with a small support set [8–10]. Optimization-based meta-learning methods learn parameters that can adapt with a few gradient updates [11,12]. Recent variants have introduced meta-transfer learning, heterogeneous-signal fusion, feature reuse, and task-supervised inner-loop optimization [13,14]. These methods improve adaptation from limited data, but the target support set remains sparse. A prototype or adaptation gradient estimated from a few target samples may describe only a small part of the target-class distribution.

Data generation addresses sample scarcity more directly. Generative adversarial networks (GANs), variational autoencoders, and Wasserstein autoencoders have been used to learn fault-data distributions and synthesize minority or scarce fault samples [15–17]. Recent GAN-based studies also show that generative augmentation can alleviate class imbalance and improve small-sample diagnosis [18–20]. The limitation is the domain in which the generated samples are used. Many generative methods augment the original training condition or a randomly partitioned dataset. The generated samples then mainly enrich the source-domain or same-condition distribution. This is not enough for cross-condition diagnosis, where the target-domain morphology may differ from the source domain.

Recent equipment-diagnosis frameworks have also moved toward graph learning, knowledge distillation, contrastive learning, and digital twins. The dual-correlation graph knowledge-distillation network (DCGKDN) models dynamic graph relations in vibration data and transfers spatiotemporal knowledge from a teacher model to a lighter student model for bearing prognostics [21]. Digital twin-assisted graph contrastive domain adaptation (GCDA) generates twin samples by coupling simulated bearing responses with measured-signal components, and then learns domain-invariant features through graph contrastive adaptation [22]. Graph convolutional domain adversarial networks (GCDAN) further combine graph convolution and adversarial adaptation for cross-domain few-shot rolling-bearing diagnosis [23]. These studies show the value of graph structure, contrastive objectives, synthetic data, and domain adaptation in modern equipment diagnosis.

The remaining problem is more specific. In target-domain few-shot cross-condition diagnosis, the labelled target classes are sparse before alignment begins. Knowledge-distillation and contrastive-learning frameworks mainly improve feature extraction or model efficiency. Digital-twin and graph-based adaptation methods improve transfer, but they do not directly test whether target-domain generative augmentation can make sparse labelled target classes more representative before class-wise alignment. Global alignment losses, such as maximum mean discrepancy (MMD), correlation alignment (CORAL), and joint distribution adaptation, reduce marginal source-target discrepancy [24–26]. They are useful when the two domains mainly differ by a global shift. Bearing faults, however, may shift in different directions under a new operating condition. A global alignment loss can reduce the overall domain gap while blurring the boundary between fault categories. Adversarial adaptation has a similar limitation. It can suppress domain-specific information [27,28], but it does not ensure that each source fault class is aligned with the corresponding target fault class.

This study therefore uses a sequential target-domain adaptation strategy. Instead of directly aligning a well-sampled source domain with a sparse target support set, the proposed framework first enriches the labelled target-class representation and then performs class-wise alignment. The vibration signal is enhanced using the rime optimization algorithm (RIME) to optimize variational mode decomposition (VMD), and the reconstructed signal is converted into a symmetric dot pattern (SDP) image. The fast generative adversarial network (FastGAN) is trained with labelled target support samples from each fault category to generate auxiliary target-domain SDP images. The generated images are used to enrich the target fault morphology under the target operating condition, rather than to augment the source domain or a mixed training set. Class-wise maximum mean discrepancy (CMMD) is then embedded into a Swin Transformer classifier to align source and target features within each known fault category.

The main contributions are as follows. First, a target-domain few-shot adaptation framework is developed that places target-domain sample enrichment before class-wise source-target alignment. Second, a signal-enhanced target-domain SDP generation strategy is built by combining RIME-optimized VMD with FastGAN-based augmentation. Third, a label-guided CMMD alignment mechanism is introduced under a strictly separated support-query protocol, allowing the effects of target-domain augmentation and class-wise alignment to be evaluated without target-query leakage.

The remainder of this paper is organized as follows. Section 2 introduces the fundamental theories used in this study, including VMD, RIME, SDP, and the FastGAN generative model. Section 3 describes the proposed method in detail, including RIME-optimized VMD, SDP parameter selection, CMMD-based feature alignment, and the overall fault diagnosis framework. Section 4 presents the experimental settings based on the CWRU and XJTU-SY rolling bearing datasets. Section 5 reports and analyses the experimental results. Section 6 discusses the mechanism, advantages, and limitations of the proposed framework. Section 7 concludes the paper.

## Materials and methods

### Variational Mode Decomposition (VMD)

The VMD algorithm possesses adaptive signal decomposition characteristics for nonlinear and nonstationary signals. Its essence is to construct a variational constraint framework with the augmented Lagrange multiplier λ(t) and the quadratic penalty factor α, decomposing the original signal f(t) into k-order modal components $\mu_{k}\left(t\right)$ and their central frequencies $\omega_{k}$, which satisfy the following constraint conditions:


$$\begin{array}{cc} L\;\left(\min_{\left\{\mu_{k}\right\},\left\{\omega_{k}\right\}},\lambda (t)\right) & =\sum_{k=1}^{K}{\left\|\begin{array}{cc} & a_{t}\left[\left(\delta (t)+\frac{j}{\pi t}\right)*\mu_{k}(t)\right]\\ & \;\times e^{-j\omega_{k}t} \end{array}\right\|}_{2}^{2}\\ & \;+{\left\|f(t)-\sum_{k=1}^{K}\mu_{k}(t)\right\|}_{2}^{2}\\ & \;+\left\langle \lambda (t),f(t)-\sum_{k=1}^{K}\mu_{k}(t)\right\rangle . \end{array}$$ (1)

In Equation (1), the penalty factor α controls the intensity of bandwidth regularization, and the number of modes *k* determines the decomposition dimension of the signal f(t). The specific process of VMD decomposition will not be elaborated here, and reference can be made to [29].

### Rime Optimization Algorithm (RIME)

In 2023, Su et al. proposed the RIME search algorithm by simulating the natural ice crystal growth process [30]. It includes three core mechanisms: soft rime random search, hard rime directional puncture, and forward greedy selection, and is suitable for solving complex optimization problems.

(a) Initialize the population

First, initialize and generate a population matrix *R*, where the population contains ice crystal individuals $x_{i}$ composed of randomly generated ice crystal particles $S_{i}$. Here, *i* denotes the ordinal number of particles, and *j* denotes the ordinal number of individuals. Therefore, the population matrix *R* containing candidate solutions can be expressed as Equation (2).


$$\begin{array}{c} R=\left[\begin{array}{c} S_{1}\\ S_{2}\\ \vdots \\ S_{i} \end{array}\right]=\left[\begin{array}{cccc} x_{11} & x_{12} & \cdots & x_{1j}\\ x_{21} & x_{21} & \cdots & x_{2j}\\ \vdots & \vdots & \ddots & \vdots \\ x_{i1} & x_{i2} & \cdots & x_{ij} \end{array}\right] \end{array}$$ (2)

(b) Soft Rime Search Strategy

The soft rime search is the global search mechanism of RIME, which continuously updates the potential optimal region by simulating the random movement of ice crystal particles to prevent the algorithm from prematurely falling into local optimal solutions. This mechanism can be expressed as Equation (3).


$$\begin{array}{cc} R_{new}^{ij} & =R_{best}^{j}+\beta \cdot \cos\theta \cdot r_{1}\left[h\cdot \left(U_{b}^{j}-L_{b}^{j}\right)+L_{b}^{j}\right]\\ {\text{r}}_{2} & <E \end{array}$$ (3)

The adhesion coefficient *E* is calculated as


$$\begin{array}{c} E(t)=\sqrt{\frac{t}{T}}, \end{array}$$ (4)

where *t* is the current iteration number and *T* is the maximum number of iterations.

$R_{new}^{ij}$ represents the new position of the particle after update, and $R_{best}^{j}$ represents the current optimal position of the particle; $U_{b}^{j}$ and $L_{b}^{j}$ represent the upper and lower bounds of the effective particle update region, respectively, and if the particle exceeds the bounds, it is clamped to the boundary; *E* represents the adhesion coefficient, which together with the random parameter *r*_2_ controls the particle update probability; parameter *r*_1_ represents the particle update direction, and its value is randomly generated within [−1,1]; *h* represents the “adhesion distance” between the particle and the current optimal solution, and its value is continuously adjusted within [0,1]. The environmental factor β and the oscillation term cosθ are shown in Equation (5). Both β and cosθ decay as the number of iterations increases, realizing the adaptive transition of particles from large-scale search to local search.


$$\begin{array}{cccc} \theta & =\frac{\pi t}{10T}, & \beta & =\frac{1}{\omega }\left|\frac{\omega \cdot t}{T}\right| \end{array}$$ (5)

(c) Hard Rime Puncture Strategy

The hard rime puncture strategy is the local exploitation mechanism of RIME. By simulating the phenomena of directional growth and random cross-penetration of ice crystal particles under strong wind, it achieves a balance between fast convergence and controllable disturbance of the algorithm. This puncture strategy can be expressed as Equation (6).


$$\begin{array}{c} R_{new}^{ij}=R_{best}^{j},\;\text{if }r_{3}<F_{norm}\left(S_{i}\right) \end{array}$$ (6)

For the minimization objective used in this study, the normalized fitness value of the *i*-th individual is calculated as


$$\begin{array}{c} F_{norm}(S_{i})=\frac{F(S_{i})-F_{\min}}{F_{\max}-F_{\min}+\varepsilon }, \end{array}$$ (7)

where $F(S_{i})$ is the fitness value of individual $S_{i}$, $F_{\min}$ and $F_{\max}$ are the minimum and maximum fitness values in the current population, respectively, and ε is a small constant used to avoid division by zero. *r*_3_ is a random trigger factor within [0, 1]. The normalized fitness value is used to perform cross-puncture on selected individuals with poor fitness and update them to the position $R_{best}^{j}$ of the j-th particle in *R*.

(d) Forward Greedy Selection Mechanism

The forward greedy selection mechanism acts as the update engine of the population, guiding it toward high-quality solutions. In the soft rime search mechanism, it employs forward greedy selection to filter out high-quality solutions. In the hard rime puncture, it ensures the correct direction of population update through the same method, thereby accelerating the algorithm’s convergence.

### Symmetric Dot Pattern (SDP) algorithm

Symmetric dot pattern (SDP) maps the one-dimensional time-series signal $f_{t}$ into symmetric point pairs in the polar coordinate system, realizing signal feature visualization in the form of mirror-symmetric snowflake plots. Therefore, SDP images can still maintain the stability of fault modes under different loads and rotational speeds, making them suitable for fault diagnosis research under multiple operating conditions. The polar coordinate transformation of SDP is shown in Equation (8):


$$\begin{array}{cc} r_{i} & =\frac{f_{i}-f_{min}}{f_{max}-f_{min}}\\ \theta_{i} & =\theta +\text{g}\cdot r_{i+L}\\ \phi_{i} & =\theta -\text{g}\cdot r_{i+L} \end{array}$$ (8)

$f_{i}$ is the i-th sampling point in the signal, and after normalization, the polar coordinate radius $r_{i}$ reflects the amplitude intensity of this point; θ is the reference angle of the symmetry axis, whose value determines the number of snowflake petals generated; *g* is the angular domain scaling factor, whose value determines the opening and closing angle of the petals; *L* is the time delay coefficient, whose value determines the correlation step size of the samplin*g* points; $r_{i+L}$ is the normalized amplitude of the future L-th point. By virtue of the dynamic correlation between the previous point and the future point, SDP overcomes the defect that traditional polar coordinate mapping ignores the time-series correlation of signals, enabling the image to contain the frequency-domain characteristics of the signal. Fig 1 shows the mirror-symmetric distribution of the signal at the sampling points.

**Fig 1:**
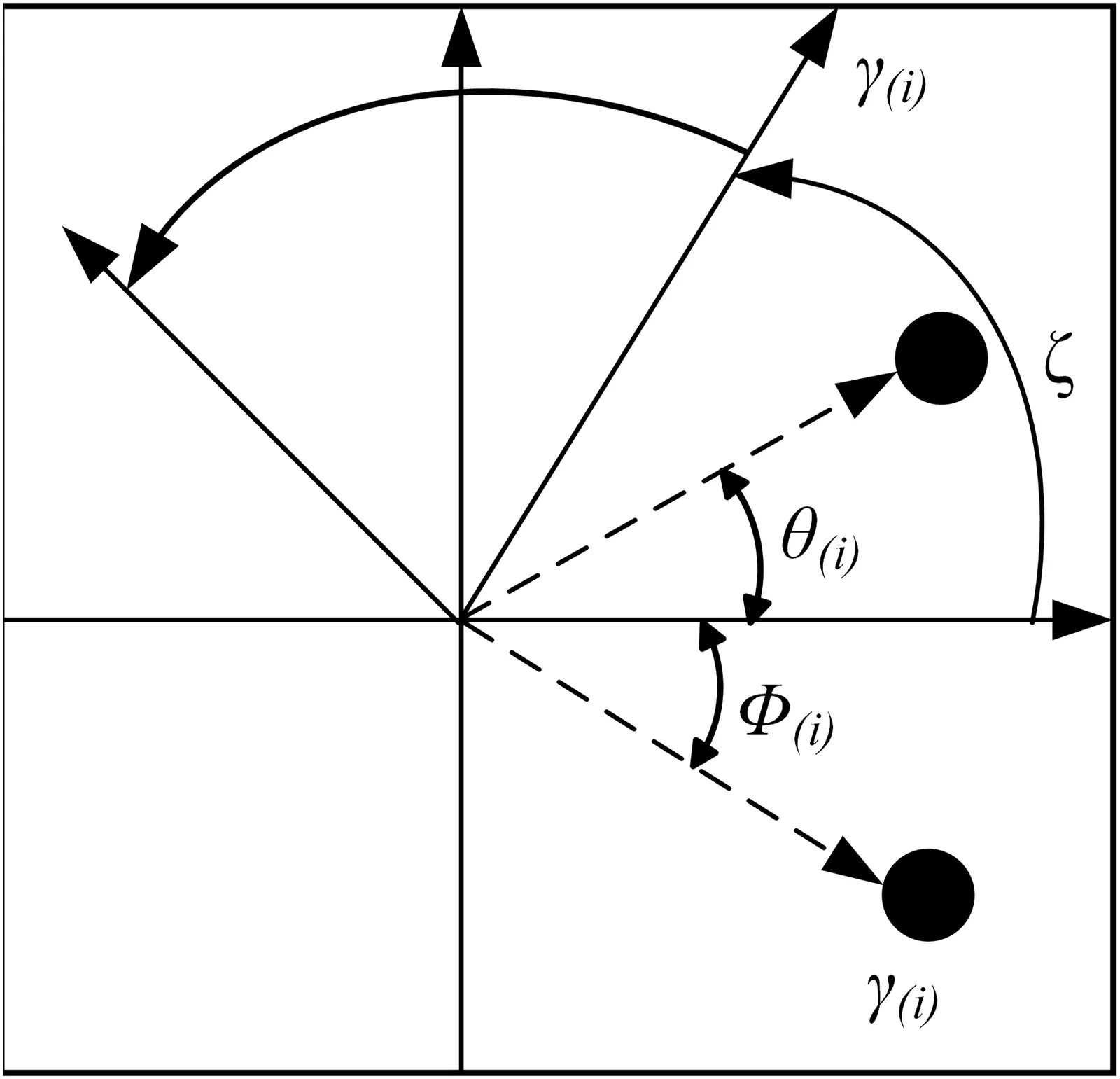
Schematic of mirror distribution of SDP [24].

### Fast Generative Adversarial Network (FastGAN) model

The fast generative adversarial network (FastGAN) is a lightweight variant of the generative adversarial network (GAN) framework designed for small image datasets. Its generator uses skip-layer channel excitation (SLE) to modulate feature maps across resolutions. SLE replaces part of the convolutional computation with channel-wise multiplication, which shortens the gradient path between low- and high-level features. This design is useful here because each target condition provides only a small labelled support set. The SLE gating operation is written as Equation (9):


$$\begin{array}{c} y=x_{high}⊙\sigma \left(W_{2}\cdot \mathrm{Leaky}\mathrm{ReLU}\left(W_{1}\cdot \mathrm{Pool}\left(x_{low}\right)\right)\right) \end{array}$$ (9)

In the formula, the low-level features are denoted as $x_{low}$, the high-level features as $x_{high}$, and the parameters to be learned as $W_{i}$. Firstly, one-dimensional noise undergoes operations such as deconvolution and normalization to obtain feature samples. The SLE gating modulation compresses the noise feature map obtained through average pooling via convolutional layers to reduce spatial dimensions. Secondly, after dimensionality reduction via a fully connected layer and activation functions, the number of channels is adjusted to ensure consistency. Finally, the function generates a channel-weight vector, which is multiplied channel-wise with the feature map to produce the output image. The generator modulates features at each resolution level through SLE and is optimized adversarially against the discriminator before being used to produce auxiliary samples. The detailed algorithm is described in Reference [31].

## Proposed methods

### RIME-Optimized Variational Mode Decomposition (RVMD)

To address the problem that the number of modes *k* and the quadratic penalty factor α in VMD decomposition cannot be adaptively determined, this paper defines a weighted objective composed of envelope entropy and the adjacent-mode spectral-overlap penalty as the RIME fitness function to optimize the VMD parameter combination. Hereafter, this method is denoted as RIME-optimized variational mode decomposition (RVMD), and the specific steps are as follows:

Step 1: Define the RIME search settings for RVMD: k∈[2,10], α∈[2000,5000], maximum number of iterations $N_{\max}=50$, and population size $R_{\max}=20$.

Step 2: Define the fitness function: In Equation (10), $H_{env}(k,\alpha )$ denotes the envelope entropy of the IMF envelope sequence obtained under the candidate VMD parameters (k,α). A lower value indicates higher signal sparsity and more obvious fault-related impulse characteristics. The symbol $p_{m}$ denotes the normalized envelope-amplitude probability at the *m*-th sampling point, *a*(*m*) denotes the envelope amplitude sequence, and *n* is the signal length.


$$\begin{array}{cc} H_{env}(k,\alpha ) & =-\sum_{m=1}^{n}p_{m}\ln p_{m}\\ p_{m} & =\frac{a(m)}{\sum_{m=1}^{n}a(m)} \end{array}$$ (10)

In Equation (11), *P* is the penalty term for adjacent-mode spectral overlap. $f_{max,k}$ denotes the upper frequency boundary of the dominant spectral band of the *k*-th IMF component, and $f_{min,k+1}$ denotes the lower frequency boundary of the dominant spectral band of the adjacent (*k* + 1)-th IMF component. When $f_{max,k}>f_{min,k+1}$, the two adjacent dominant frequency bands overlap, indicating modal aliasing, and the overlap width is penalized. When $f_{max,k}\leq f_{min,k+1}$, no adjacent-band overlap is detected and the penalty term is 0. Therefore, the weighted objective J(k,α) combines envelope entropy and adjacent-mode spectral-overlap penalty to guide the balance between modal sparsity and spectral separation.


$$\begin{array}{cc} P & =\sum_{k=1}^{K-1}\max\left(0,f_{max,k}-f_{min,k+1}\right)\\ J(k,\alpha ) & =0.8H_{env}(k,\alpha )+0.2P \end{array}$$ (11)

Step 3: Input the vibration signal f(x) and initialize the ice crystal population *R*.

Step 4: Calculate the adhesion coefficient *E* according to Equation (4). Update the particle positions using Equation (3), calculate the normalized fitness value $F_{norm}(S_{i})$ according to Equation (7), and continue updating the particle positions if the random value *r*_3_ satisfies the condition in Equation (6).

Step 5: Compare the fitness function values of the particles before and after update. Replace the position if the new value is better than the previous one; otherwise, repeat Step 4.

Step 6: When the iteration meets the termination condition of $N_{\max}=50$, output the optimal parameter combination [k,α].

### SDP parameter selection criteria

The Pearson correlation coefficient is used here to measure the linear dependence between flattened SDP images generated under different parameter settings. Compared with visual similarity measures such as the Structural Similarity Index Measure and the Perceptual Hashing Algorithm, correlation-based evaluation is more directly tied to the numerical distribution of the transformed vibration signal. For each candidate parameter setting, Pearson correlations are calculated between SDP image pairs from different training fault classes and then averaged to obtain the inter-class correlation. Validation, test, and target-query samples are not used for SDP parameter selection. In this study, the parameter setting with lower positive inter-class image correlation is selected so that SDP images from different fault types retain clearer differences.


$$\begin{array}{c} \rho =\frac{\sum_{i=1}^{n}\left(x_{i}-\bar{x}\right)\left(y_{i}-\bar{y}\right)}{\sqrt{\sum_{i=1}^{n}{\left(x_{i}-\bar{x}\right)}^{2}}\sqrt{\sum_{i=1}^{n}{\left(y_{i}-\bar{y}\right)}^{2}}} \end{array}$$ (12)

In Equation (12), ρ is the Pearson correlation coefficient, whose value ranges from −1 to 1. A value close to 1 indicates strong positive linear correlation between two flattened SDP images, a value close to −1 indicates strong negative linear correlation, and a value close to 0 indicates weak linear correlation. $x_{i}$ and $y_{i}$ are the pixel values at the same flattened position in two SDP images, $\bar{x}$ and $\bar{y}$ are their mean pixel values, and *n* is the total number of pixels.

### Category-constrained Maximum Mean Discrepancy (MMD)-based cross-condition feature alignment

In cross-domain diagnosis tasks, feature distribution discrepancy exists between source and target domain samples due to differences in rotational speed, load, and individual bearing characteristics. Therefore, this paper introduces the class-wise maximum mean discrepancy (CMMD) loss in target-domain few-shot adaptation experiments to align the feature distributions of the same fault type across the source and target domains. Maximum mean discrepancy (MMD) is a method for measuring distribution discrepancy, as shown in Equation (13):


$$\begin{array}{cc} {\mathrm{MMD}}^{2}\left(F_{s},F_{t}\right) & =\|\frac{1}{n_{s}}\sum_{i=1}^{n_{s}}\phi \left(f_{i}^{s}\right)\\ & \;{-\frac{1}{n_{t}}\sum_{j=1}^{n_{t}}\phi \left(f_{j}^{t}\right)\|}_{H}^{2} \end{array}$$ (13)

In the formula, $f_{i}^{s}$and $f_{j}^{t}$ denote the feature vectors extracted from the source and target domains by Swin-T, respectively. ϕ(·) represents the mapping kernel function, and H denotes the reproducing kernel Hilbert space. A smaller MMD value indicates that the feature distributions between the two domains are closer. On this basis, this paper calculates the distribution discrepancy between the source and target domains for each fault category separately, as shown in Equation (14):


$$\begin{array}{c} {ℒ}_{CMMD}=\frac{1}{C}\sum_{c=1}^{C}{\mathrm{MMD}}^{2}\left(F_{s}^{c},F_{t}^{c}\right) \end{array}$$ (14)

where C is the number of fault categories, $F_{s}^{c}$and $F_{t}^{c}$ denote the feature sets of the C-th fault category in the two domains, respectively. Therefore, the classification loss of this paper is defined as shown in Equation (15)


$$\begin{array}{c} ℒ={ℒ}_{CE}^{s}+\alpha {ℒ}_{CE}^{t}+\beta {ℒ}_{CMMD} \end{array}$$ (15)

where ${ℒ}_{CE}^{s}$ denotes the classification loss in the source domain, ${ℒ}_{CE}^{t}$ denotes the classification loss calculated from the labelled target-support samples and the generated auxiliary target samples, and α and β are the corresponding weighting coefficients. By jointly optimizing the classification loss and the CMMD loss, the model preserves fault-discrimination ability while reducing the distribution discrepancy between source and target domains. This is the basis for target-domain adaptation in the classifier.

### Problem formulation and optimization objective

The target-domain few-shot cross-condition diagnosis task is formulated as a labelled source-domain learning problem with sparse labelled target-domain adaptation. Let ${𝒟}_{s}=\{(x_{i}^{s},y_{i}^{s}{\}}_{i=1}^{N_{s}}$ denote the source-domain SDP images and labels, and let ${𝒟}_{t}^{sup}=\{(x_{i}^{t},y_{i}^{t}{\}}_{i=1}^{N_{t}}$ denote the labelled target-support set. The target-query set ${𝒟}_{t}^{q}$ is kept independent and is used only for final evaluation. In this setting, $N_{t}$ is much smaller than $N_{s}$, and the same fault category may have different feature distributions under different operating conditions:


$$\begin{array}{c} P_{s}\left(f_{\Theta }(x)|y=c\right)\neq P_{t}\left(f_{\Theta }(x)|y=c\right), \end{array}$$ (16)

where $f_{\Theta }(\cdot )$ denotes the feature extractor of the Swin-T backbone and *c* is a fault category.

The proposed framework models target sparsity and class-conditional distribution mismatch in a coupled manner. FastGAN is trained using the labelled target-support samples and generates an auxiliary target-domain set ${\tilde{𝒟}}_{t}$. The target set used for downstream adaptation is therefore


$$\begin{array}{c} {𝒟}_{t}^{aug}={𝒟}_{t}^{sup}\cup {\tilde{𝒟}}_{t}. \end{array}$$ (17)

The classifier is then optimized using the unified objective in Equation (15), which combines source-domain classification, augmented target-domain classification, and CMMD-based class-wise feature alignment.

This formulation links the two main challenges of the task. The generated target-domain samples increase the effective sample size of each target class and stabilize the estimation of the target class-wise feature distribution. The CMMD term then aligns the source-domain features with the augmented target-domain features within each fault category. Therefore, target sparsity is incorporated through ${𝒟}_{t}^{aug}$, while class-conditional offset is constrained through ${ℒ}_{CMMD}$. The whole framework follows a staged optimization strategy: RVMD and SDP provide signal-enhanced image representations, FastGAN expands the sparse target-support distribution, and Swin-T is trained with the classification-alignment objective for target-domain adaptation.

### Fault diagnosis framework

Fig 2 shows the proposed fault diagnosis framework. In the signal-processing stage, VMD is replaced by RVMD following the procedure in Section 3.1, which reduces the dependence of denoising quality on manually selected decomposition parameters. After multi-scale decomposition, IMF components with kurtosis values above the average are used to reconstruct the vibration signal. The reconstructed signal is then mapped into a two-dimensional mirror-symmetric SDP image. In this representation, petal shape and point density reflect amplitude and temporal variation in the vibration signal. For data augmentation, FastGAN is trained on the available SDP images under the corresponding target condition. The discriminator treats real SDP images as reference samples, while the generator maps one-dimensional random noise into auxiliary SDP images. The generated samples are then used to expand the limited target support set for classifier adaptation.

**Fig 2:**
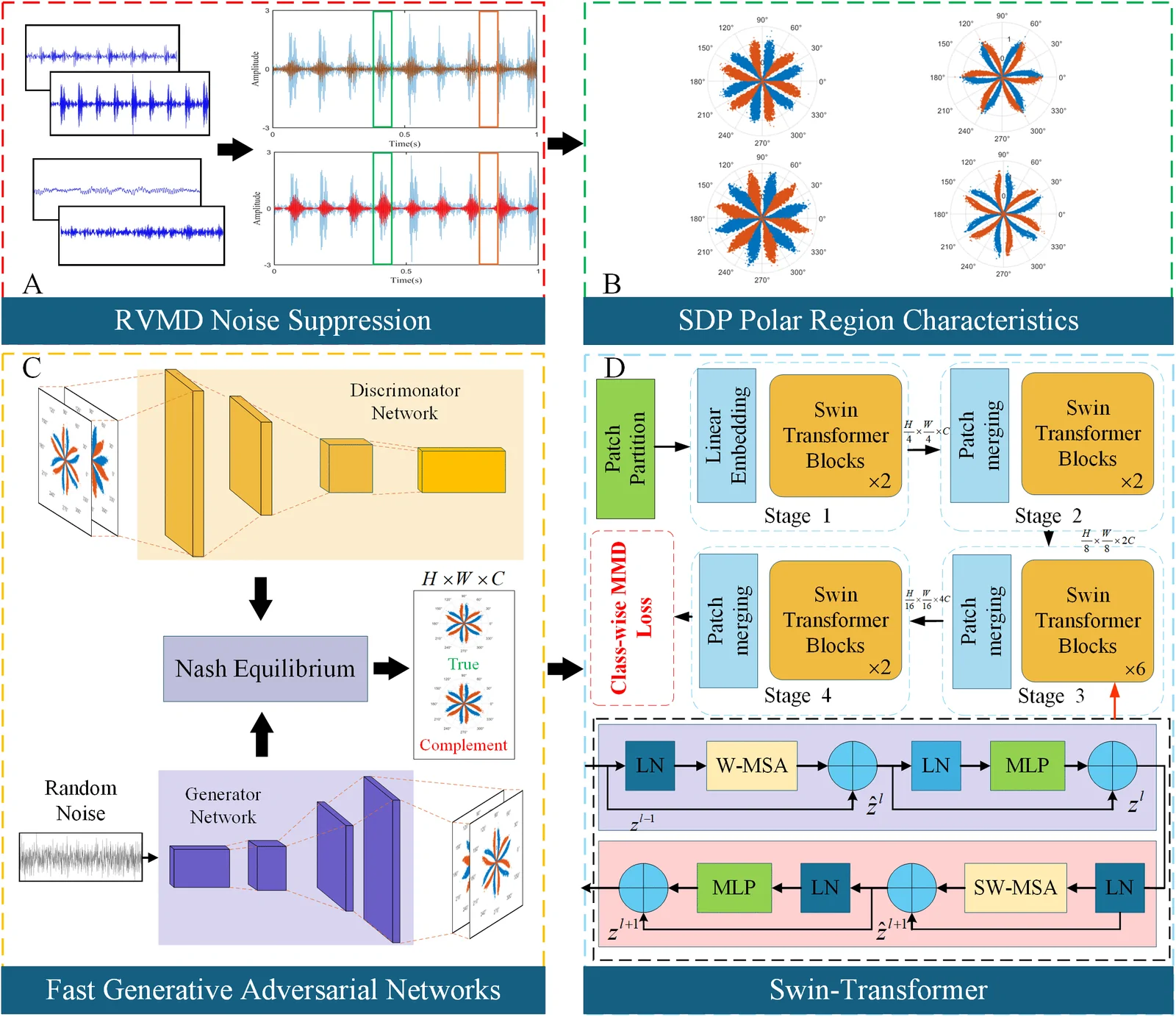
Proposed fault diagnosis framework.

The classifier is built on Swin Transformer (Swin-T) because SDP images contain both local texture patterns and global polar structures. A conventional convolutional model mainly aggregates local neighborhoods, whereas Swin-T can learn hierarchical image features through window-based self-attention. The patch partition layer first splits each SDP image into non-overlapping 4×4 patches and projects them into token embeddings. The subsequent stages use Window Multi-Head Self-Attention (W-MSA) to model local dependencies within fixed windows and Shifted Window Multi-Head Self-Attention (SW-MSA) to exchange information across neighboring windows. Patch merging between stages reduces spatial resolution and increases channel dimension, forming multi-scale features from detailed local patterns to higher-level fault morphology.

This feature hierarchy provides the interface between Swin-T and the CMMD loss defined in Section 3.3. During training, real source-domain SDP images, labelled target-support SDP images, and FastGAN-generated target-domain SDP images are all passed through the same Swin-T backbone. The feature vector before the final fully connected classifier is used for two purposes: cross-entropy loss preserves fault-discriminative classification, while CMMD aligns source and target feature distributions separately within each fault category. In this way, Swin-T supplies the shared representation space, FastGAN reduces the sparsity of the target support distribution, and CMMD constrains class-wise source-target mismatch in that representation space. Table 1 summarizes the Swin-T configuration used in this framework and indicates the feature output used for class-wise alignment.

**Table 1:** Swin-T configuration and feature interface for CMMD alignment.

Item	Stage 1	Stage 2	Stage 3	Stage 4
Blocks	2	2	6	2
Embedding dimension	96	192	384	768
Attention heads	3	6	12	24
Window size	7×7	7×7	7×7	7×7
Output feature map	56×56×96	28×28×192	14×14×384	7×7×768
CMMD interface	The feature after Stage 4 and global pooling is used for cross-entropy classification and class-wise CMMD alignment.			

### Experimental validation

Two datasets were used for validation. Case 1 used the Case Western Reserve University (CWRU) dataset to examine RVMD denoising, SDP feature construction, FastGAN auxiliary-sample generation, Swin-T hyperparameter selection, and t-SNE visualization. Case 2 used the Xi’an Jiaotong University–Shenyang Blower Works (XJTU-SY) dataset to test cross-condition few-shot adaptation under a more pronounced operating-condition shift. The experimental workflow is shown in Fig 3. All experiments were run on an Intel Core i7-12700F CPU and an NVIDIA GeForce RTX 4060 Ti GPU, using Python 3.9 and PyTorch 2.3.1 with CUDA 11.2.

**Fig 3:**
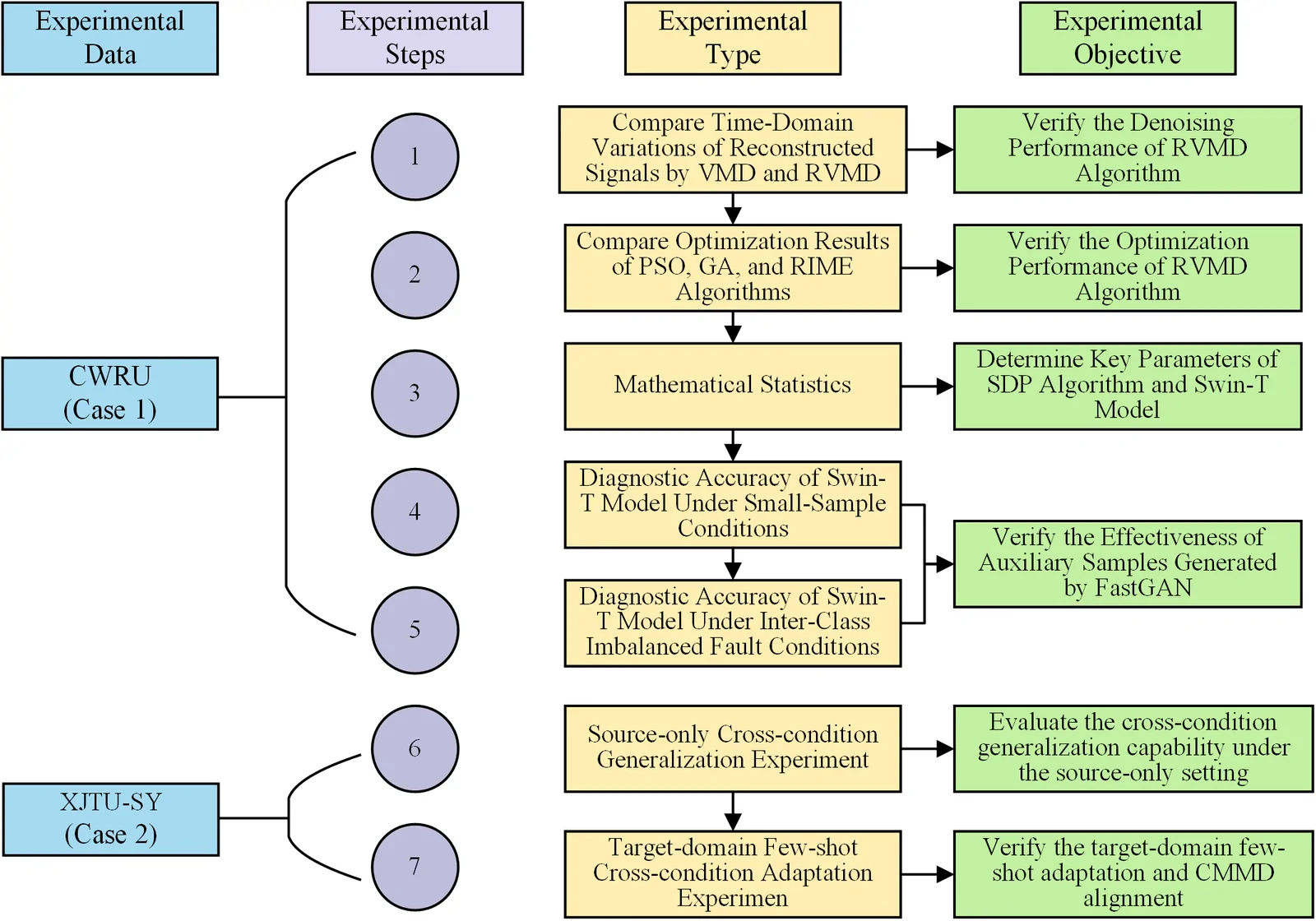
Experimental procedure and methodology.

### Case study 1

The CWRU bearing test platform comprises a motor, a dynamometer, an acceleration sensor, and test bearings installed at the fan end and drive end. The bearing models at the fan end and drive end are SKF6203 and SKF6205, respectively. Detailed parameters of the platform are available in Reference [32]. In this study, the drive-end bearing vibration signals were selected, with motor loads ranging from 0 to 3 HP, a sampling frequency of 12 kHz, and a fault diameter of 0.1778 mm. As shown in Table 2, ten classes covering four health states were used, including inner race fault (IR), outer race fault (OR), ball fault (BO), and normal state (N). For example, IR_1 denotes the inner race fault signal collected under a motor load of 1 HP with a fault diameter of 0.1778 mm, and the other fault labels are defined in the same manner.

**Table 2:** Fault types of SKF6205 bearings.

Sample Label	Motor Speed (r/min)	Motor Load (Hp)	Fault Type
0	1797	0	Normal State (N)
1	1797	0	Inner Race (IR_0)
2	1797	0	Outer Race (OR_0)
3	1797	0	Ball (BO_0)
4	1772	1	Inner Race (IR_1)
5	1772	1	Outer Race (OR_1)
6	1772	1	Ball (BO_1)
7	1750	2	Inner Race (IR_2)
8	1750	2	Outer Race (OR_2)
9	1750	2	Ball (BO_2)

Fig 4 displays the time-domain waveforms representing the four operational health states of the bearing in Case 1. The figure shows that the waveform in Subfigure (a) is relatively sparse. In contrast, the waveforms in the other subfigures are denser, with noise glitches caused by fault impacts and high-frequency oscillations, making it challenging to identify the specific bearing fault location directly.

**Fig 4:**
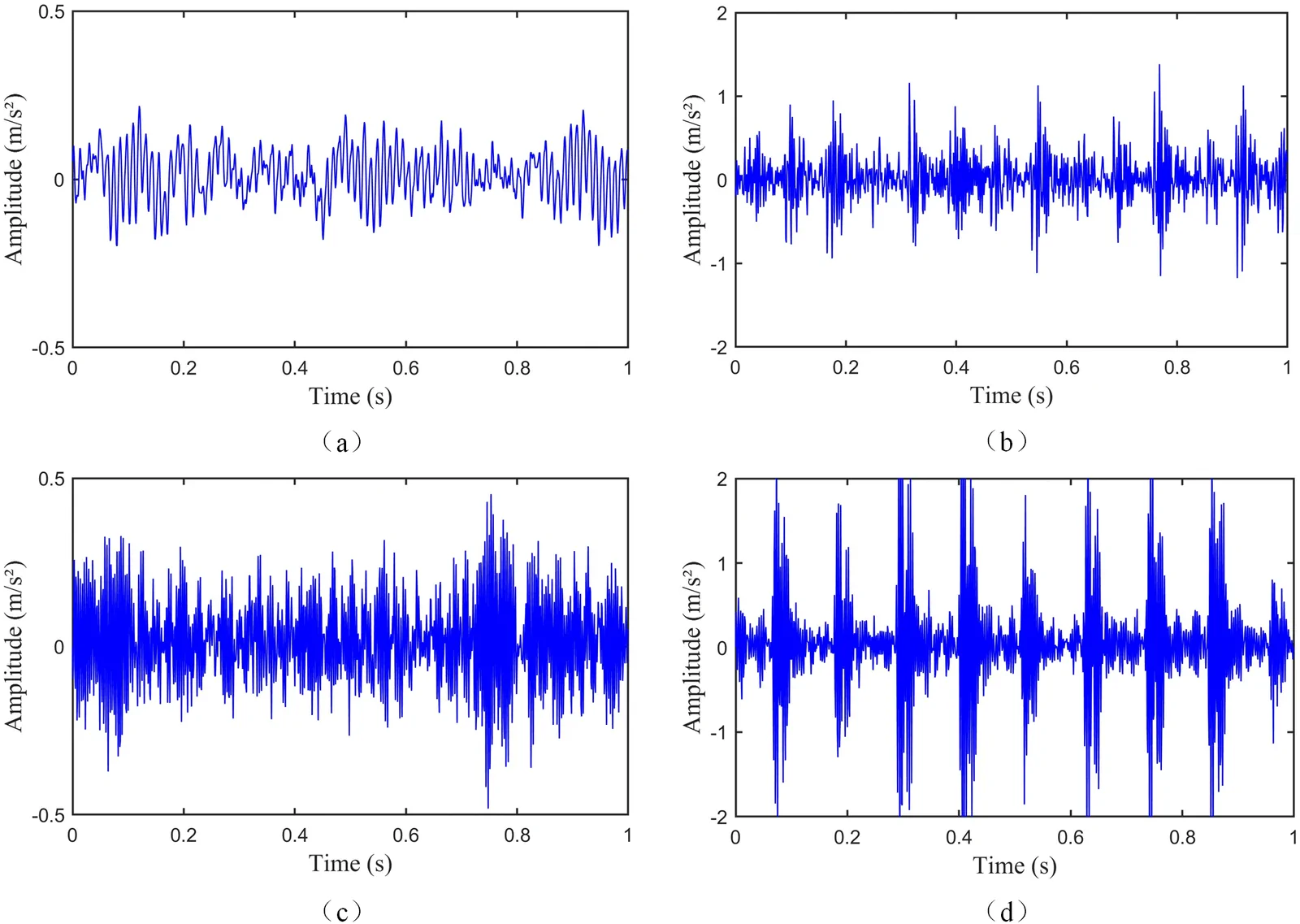
Time-domain signals of four fault types in case study 1: (a) Normal signal, (b) Inner race fault, (c) Rolling element fault, (d) Outer race fault.

### Case study 2

Case 2 used full-life-cycle bearing vibration data from the XJTU-SY dataset [33]. Its acquisition platform includes an alternator, a motor speed controller, a rotating shaft, a hydraulic loading system, support bearings, and test bearings. The hydraulic system and speed controller set the radial force and rotational speed, thereby creating different operating conditions. Vertical and horizontal acceleration sensors mounted around the test bearing collect vibration signals, and a digital force display records the applied load during operation.

This paper uses the full-life-cycle vibration signals of LDK UER204 rolling bearings as the research object. Model generalization experiments are conducted with a sampling frequency of 25.6 kHz and a sampling interval of 1 minute. Signals from the middle section of the inner race, outer race, and cage are extracted separately at rotational speeds of 2100 r/min, 2250 r/min, and 2400 r/min. The specific types and labels of the collected data are shown in Table 3.

**Table 3:** Fault types of LDK UER204 bearings.

Sample Label	Rotating Speed (r/min)	Radial Force (KN)	Fault Type
0	2100	12	Outer Race (OR_1)
1	2100	12	Cage (CF_1)
2	2250	11	Inner Race (IR_1)
3	2250	11	Cage (CF_2)
4	2250	11	Outer Race (OR_2)
5	2400	10	Inner Race (IR_2)

Fig 5 shows the time-domain waveforms for the three bearing states used in Case 2. The waveforms differ in amplitude, impact behavior, and energy distribution because the operating conditions and fault locations are different. Even so, the fault type is difficult to identify directly from the raw waveform. Baseline fluctuations and non-impact noise further obscure the fault-related components.

**Fig 5:**
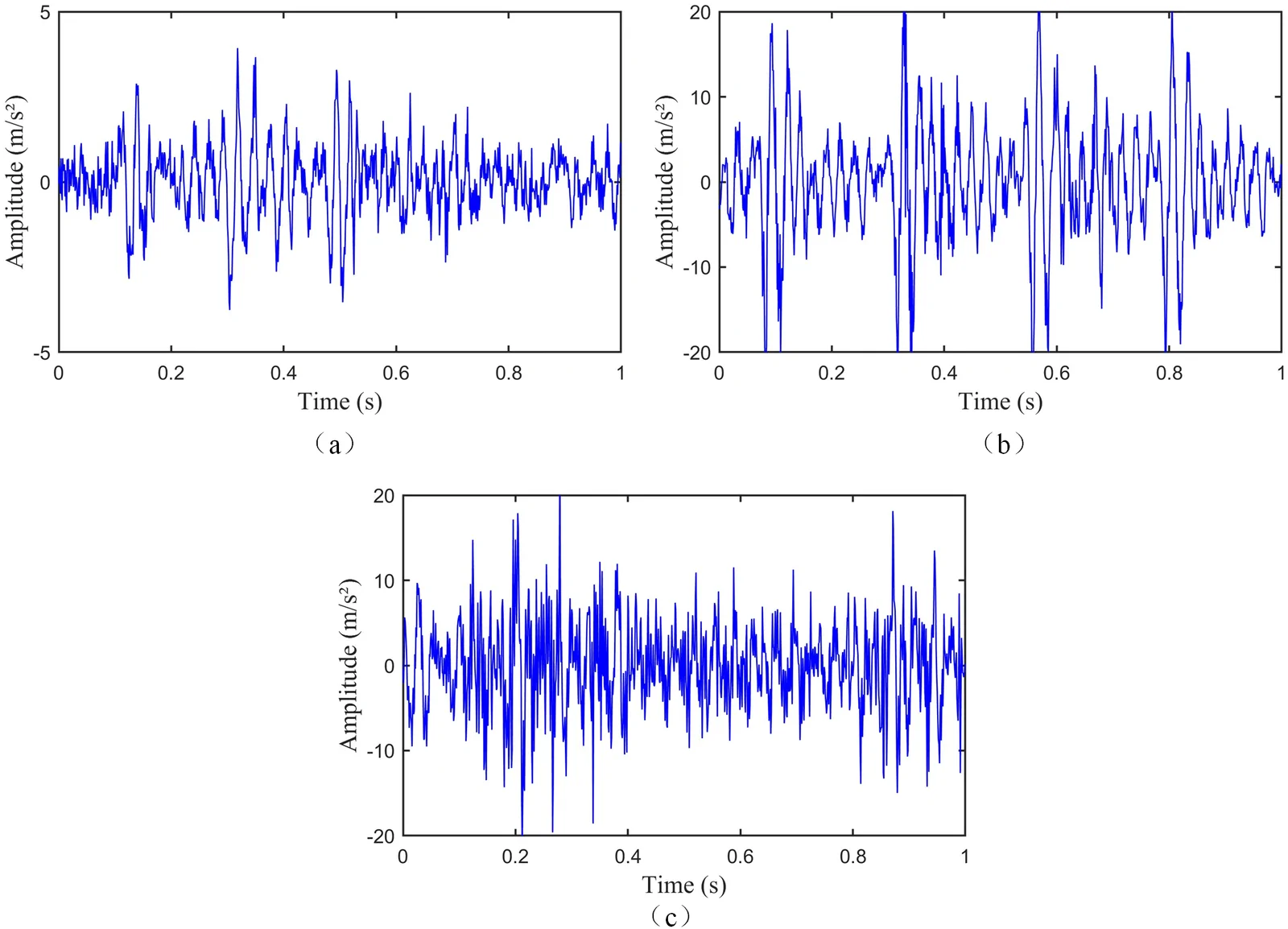
Time-domain signals of three fault types in case study 2: (a) Cage Fault, (b) Outer Race Fault, (c) Inner Race Fault.

## Results and analysis

### Case study 1

#### Experiment on RVMD signal enhancement.

The OR_1 vibration signal was used to examine the RVMD optimization procedure. RVMD parameters were configured according to Section 2.3, and the optimal parameter combination was [*k*,*a*] = [7,4701]. For comparison, VMD was also run with the parameter combination [2,2000] reported in Reference [34]. For both VMD and RVMD, IMF components with kurtosis values above the average were selected for signal reconstruction. The reconstructed time-domain waveforms are shown in Fig 6.

**Fig 6:**
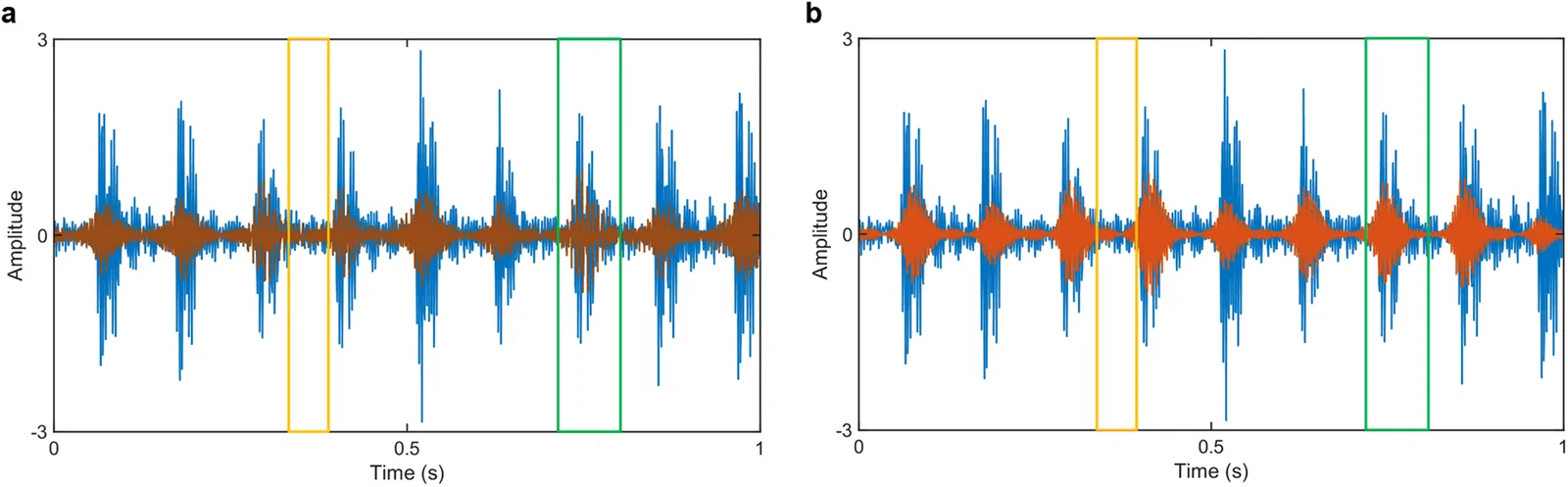
Time-domain signal superposition: (a) original signal and VMD reconstructed signal; (b) original signal and RVMD reconstructed signal.

In Fig 6, the blue curve represents the original measured signal, and the brown and red curves represent the reconstructed signals obtained by VMD and RVMD, respectively. The green region marks a fault-impact segment, and the orange region marks a segment with strong high-frequency fluctuations. Compared with the VMD reconstruction in Fig 6(a), the RVMD reconstruction in Fig 6(b) preserves sharper impact edges and produces a smoother baseline. This visual comparison indicates that RIME-based parameter optimization improves the denoising-oriented reconstruction process by suppressing residual fluctuations while retaining the main fault-impact morphology.

Root Mean Square Error (RMSE), Correlation Coefficient (CC), Signal-to-Noise Ratio (SNR), Normalized Cross-Correlation Coefficient (NCC), and Kurtosis were further calculated to characterize the reconstructed signals. Here, SNR was calculated as a relative reconstruction index using the power ratio between the reconstructed signal and the residual component between the measured and reconstructed signals. The quantitative results are shown in Table 4.

**Table 4:** Descriptive reconstruction-quality indicators of VMD and RVMD.

Method	RMSE	CC	SNR/dB	NCC	Kurtosis
VMD	0.5123	0.5634	1.4375	0.5632	5.3006
RVMD	**0.2015**	**0.8278**	**2.9690**	**0.8278**	**7.7485**

The quantitative indicators show the same trend as the waveform comparison. Compared with VMD, RVMD reduces RMSE to 0.2015, increases CC and NCC to 0.8278, and improves the SNR-related value by 1.53 dB. These results indicate that the RVMD reconstructed signal remains closer to the main measured fault-amplitude pattern and retains stronger impulsive characteristics, as reflected by the higher kurtosis value.

Spectral analysis was then used to connect the frequency-domain characteristics of the reconstructed vibration signal with the bearing fault mechanism. In this experiment, the high-frequency resonance band was identified from the power spectrum, and the envelope spectrum of the RVMD reconstructed signal was plotted, as shown in Fig 7.

**Fig 7:**
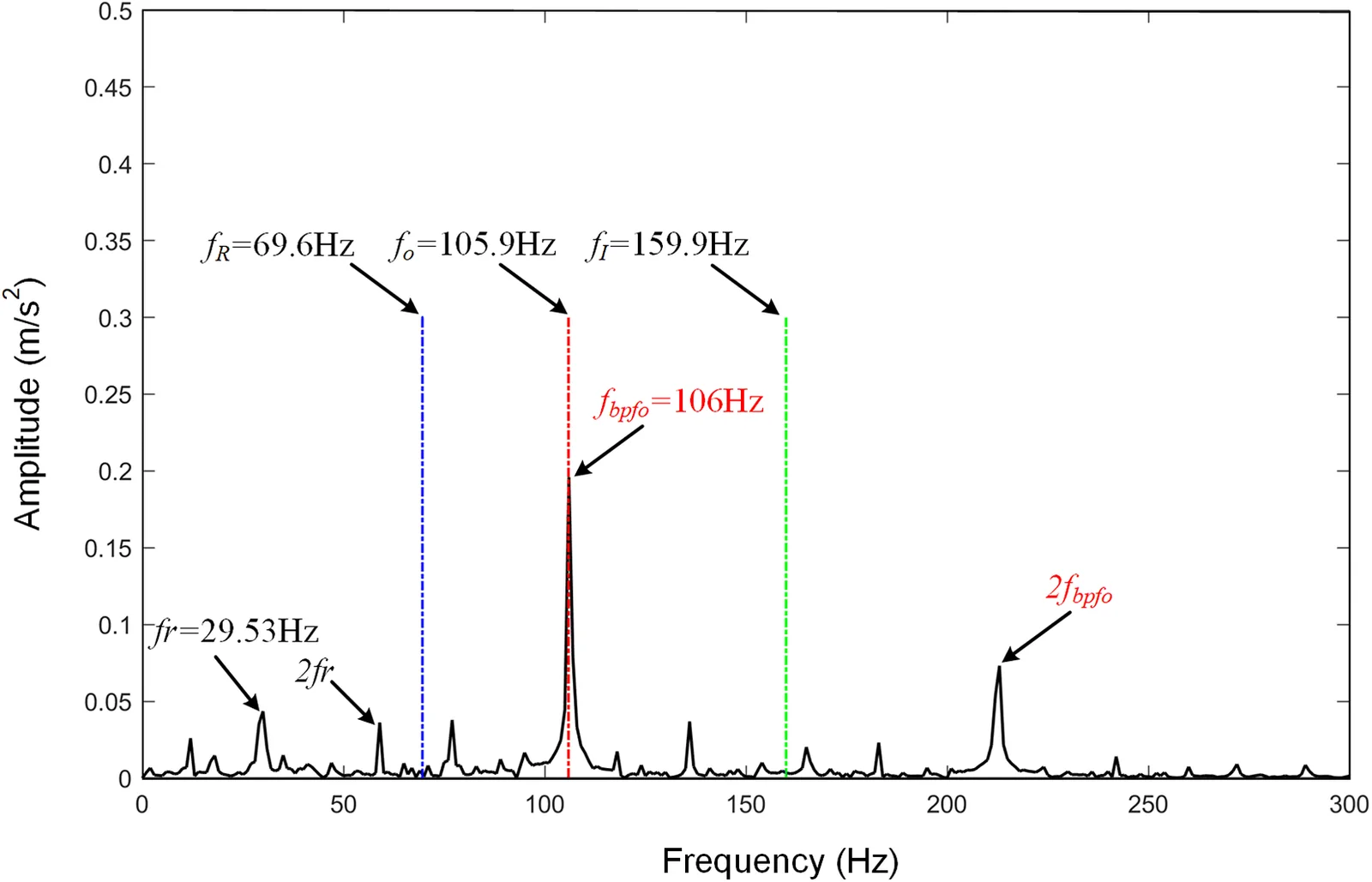
Envelope spectrum of reconstructed signal by RVMD.

Here, $f_{R}=69.6\mathrm{Hz}$, $f_{O}=105.9\mathrm{Hz}$, and $f_{I}=159.9\mathrm{Hz}$ are the theoretical fault characteristic frequencies of the ball, outer race, and inner race, respectively. In the envelope spectrum, the measured rotational frequency is 30 Hz and the measured outer-race fault frequency is $f_{bpfo}=106\mathrm{Hz}$. These values match the theoretical rotational frequency $f_{r}=29.5\mathrm{Hz}$ and outer-race fault frequency $f_{O}=105.9\mathrm{Hz}$, so the signal corresponds to an outer-race bearing fault. These time-domain and frequency-domain results show that RVMD gives a cleaner reconstructed signal than the original VMD setting and that the adaptive parameter selection is consistent with the observed fault-frequency information.

#### Comparative experiment of optimization algorithms.

RIME was compared with particle swarm optimization (PSO) and the genetic algorithm (GA) under the same optimization ranges for $N_{\max}$, $R_{\max}$, and [k,α]. For PSO, the inertia weight $\left[w_{start},w_{end}\right]$ was set to [0.9,0.3], and the learning factor *c* was set to 1.5. For GA, the crossover probability $P_{c}$ was set to 0.7, and the mutation probability $P_{m}$ was set to 0.2. All three algorithms minimized the weighted objective composed of envelope entropy and the adjacent modal-frequency overlap penalty. Fig 8 shows the iteration curves, and Table 5 lists the resulting parameters.

**Table 5:** Comparison of optimization algorithms.

Methods	Convergence Rate	Minimum Fitness	Mean Fitness	Time/s
GA	4	12.753	12.981	161
PSO	7	11.551	11.893	217
RIME	**6**	**11.420**	**11.671**	**167**

**Fig 8:**
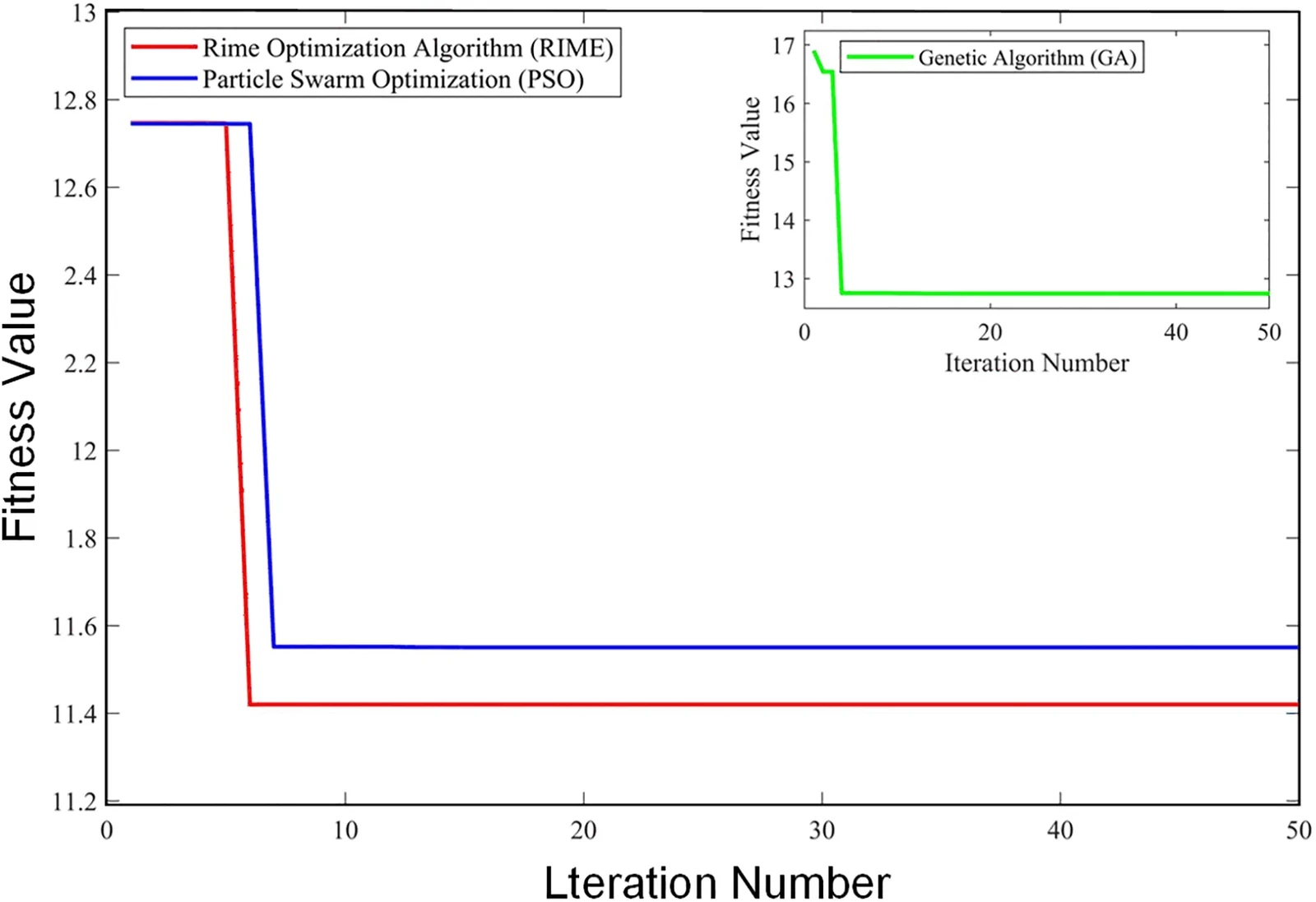
Comparison of iteration processes across different optimization algorithms.

RIME converges faster than PSO in this experiment and obtains a lower final fitness value than both GA and PSO. The result is consistent with its combined global search and local refinement behavior. In this RVMD setting, RIME therefore provides a better balance between convergence speed and denoising-oriented parameter selection.

#### SDP parameter setting and sample generation.

RVMD decomposition separates fault-related components from background noise in the time-frequency domain. SDP mapping then encodes time-domain variation into a polar-coordinate structure through the relationships between angle and time, and between radial distance and amplitude. This mapping gives each fault signal a compact spatial representation in the polar domain. In the polar coordinate space, the symmetry-axis reference angle θ, angular-domain scaling factor *g*, and time-delay coefficient *L* determine the shape of the SDP image. Previous bearin*g*-diagnosis studies have used SDP images to represent vibration-signal morphology and time-frequency variation [24,25]. Here, θ was set to $60^{\circ }$, *g* was initialized at 20 with a step size of 5, and *L* was initialized at 1 with a step size of 1. A total of 70 random parameter combinations were tested. The resulting average Pearson correlation coefficients $\bar{\rho }$ are listed in Table 6.

**Table 6:** $\bar{\rho }$ values for different parameter combinations of SDP images.

$\bar{\rho }$	*L* = 1	*L* = 2	*L* = 3	*L* = 4	*L* = 5	*L* = 6	*L* = 7	*L* = 8	*L* = 9	*L* = 10
$g=20^{\circ }$	0.6701	0.6294	0.6823	0.6970	0.6136	0.6750	0.6319	0.6833	0.6539	0.6457
$g=25^{\circ }$	0.6473	0.5997	0.6572	0.6785	0.5876	0.6563	0.6321	0.6544	0.6372	0.6193
$g=30^{\circ }$	0.6284	0.5823	0.6516	0.6541	0.5807	0.6387	0.6114	0.6401	0.6241	0.5973
$g=35^{\circ }$	0.6421	0.5796	0.6622	0.6662	**0.5661**	0.6369	0.6014	0.6406	0.6254	0.5974
$g=40^{\circ }$	0.6753	0.5794	0.6589	0.6841	0.5858	0.6578	0.6304	0.6567	0.6420	0.6270
$g=45^{\circ }$	0.6743	0.6010	0.6821	0.7040	0.5989	0.6613	0.6362	0.6758	0.6730	0.6471
$g=50^{\circ }$	0.6806	0.6376	0.6877	0.6985	0.6302	0.6795	0.6602	0.6872	0.6869	0.6637

The lowest average inter-class Pearson correlation coefficient, $\bar{\rho }=0.5661$, is obtained when $g=35^{\;\;\;{\;}^{\circ }\;\;\;}$ and *L* = 5. Under this parameter combination, the training SDP images of the 10 fault types show lower linear similarity and better separability.

#### Experiment on Swin-T parameter setting.

Batch size (Batch_size) and learning rate (*Lr*) affect convergence and diagnostic accuracy during Swin-T training. When *Lr* was set to 0.001 or higher in preliminary tests, the model showed low accuracy and unstable convergence. For the multi-condition small-sample setting in this study, *Lr* was therefore fixed at 0.0001. Five batch sizes, 4, 8, 16, 32, and 64, were then compared. Fig 9 shows the Swin-T training loss curves within 100 epochs under these settings.

**Fig 9:**
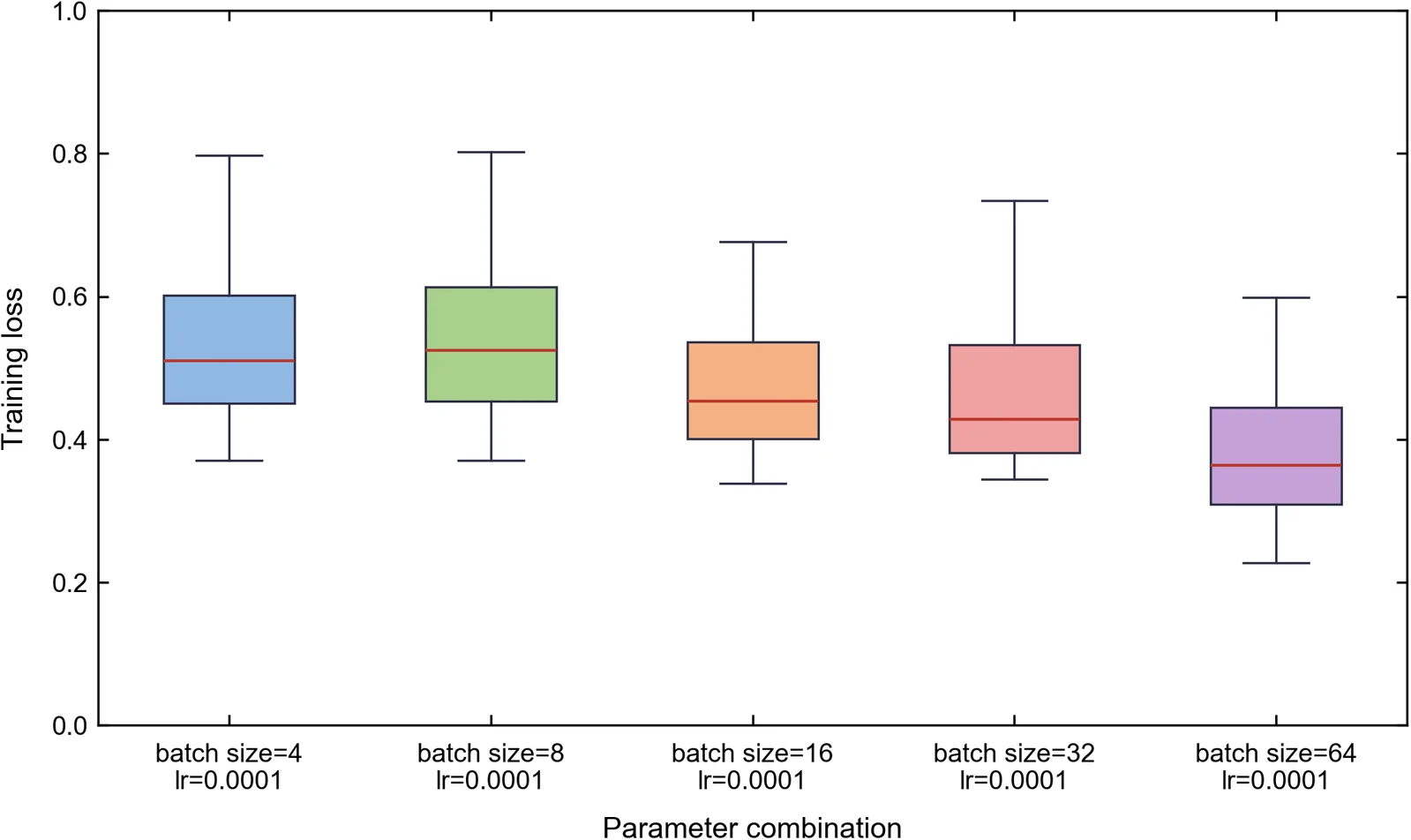
Training loss of the Swin-T model under different parameter combinations.

The boxplot shows that the median training loss decreases as the batch size increases. At Batch_size=64, the interquartile range and median value are 0.1353 and 0.3639, respectively, and the whiskers are shorter than those of the other settings. A larger batch size gives a more stable gradient estimate in each iteration, which reduces loss fluctuation during training. Therefore, Batch_size=64 and *Lr* = 0.0001 were used for the Swin-T model in the following experiments.

#### FastGAN generated auxiliary samples.

In this subsection, the SDP images of the 10 bearing states listed in Table 2 were used to evaluate FastGAN-based auxiliary-sample generation under limited-data conditions. Before model training, the real SDP images were divided into mutually exclusive training and test subsets. For each fault class, only 20 real SDP images from the training subset were used to train an independent class-specific FastGAN generator. The test images were not used for FastGAN training, generated-sample selection, classifier training, or parameter tuning, and were reserved only for final performance evaluation. The FastGAN-generated images were added only to the downstream classifier training set.

FastGAN was optimized with Adam, with learning rates of 0.0002 for both the generator and discriminator, a batch size of 4, and a latent dimension of 128. To improve training stability in the 20-sample setting, DiffAugment, spectral normalization, lazy R1 regularization, exponential moving averaging of the generator, and skip-layer channel-excitation connections were used. Each class-specific model was trained for 2500 optimization steps and generated 120 auxiliary SDP images. Figure 10 compares representative real and generated samples. The generated images retain the main petal shape, curvature, and scatter distribution of their corresponding real classes. Their quality was subsequently evaluated using FID, image-entropy difference, and downstream diagnostic performance.

**Fig 10:**
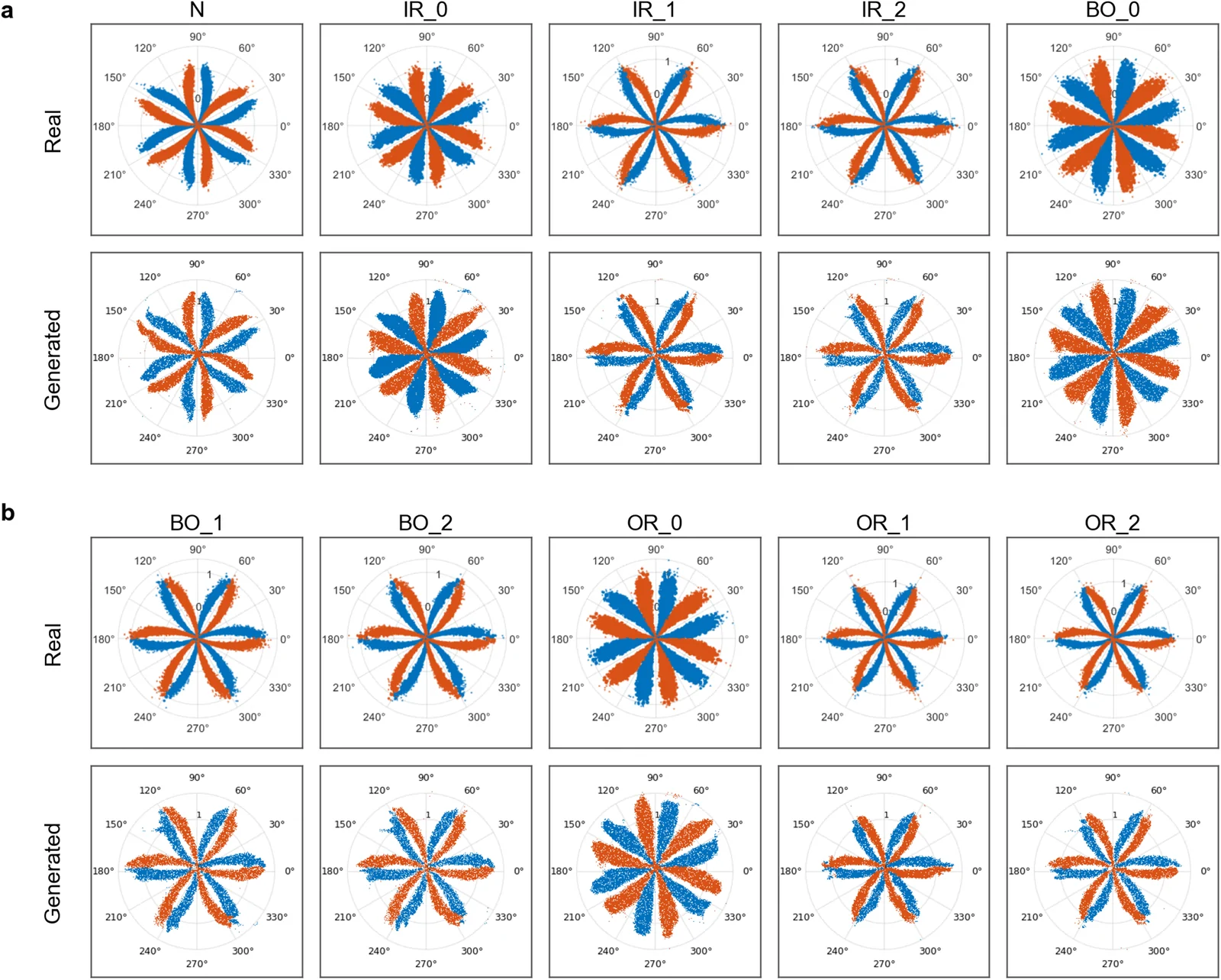
Representative real and FastGAN-generated SDP image pairs for the 10 bearing states. (a) Normal, inner-race, and ball-fault states at the first operating condition. (b) Ball- and outer-race-fault states at the remaining operating conditions.

For the controlled image-quality comparison, the deep convolutional generative adversarial network (DCGAN), Wasserstein generative adversarial network (WGAN), and FastGAN were each trained independently using five random seeds. Each run used 20 real SDP images per class, 2000 generator-update steps, a batch size of 16, and a latent dimension of 128. Each model generated 100 images per class, of which 20 generated images per class were used to calculate the Fréchet Inception Distance (FID) and the SDP image entropy difference, denoted as ΔH, matching the number of real images used for evaluation. Results are reported as mean ± standard deviation. FID measures feature-space discrepancy between generated and real images, whereas ΔH measures the difference in image-information complexity. Table 7 shows that FastGAN achieves the lowest FID and ΔH values for all four fault groups.

**Table 7:** Evaluation of SDP image generation using five independent training runs (mean ± standard deviation).

Fault Type	DCGAN		WGAN		FastGAN	
	FID	ΔH	FID	ΔH	FID	ΔH
Inner Race	186.61±11.16	0.460±0.007	219.43±27.03	0.490±0.010	** 123.98±11.64 **	** 0.381±0.003 **
Outer Race	184.65±6.34	0.448±0.006	213.58±27.17	0.487±0.011	** 124.41±7.18 **	** 0.368±0.005 **
Ball	191.74±6.00	0.468±0.006	215.72±28.06	0.505±0.009	** 132.27±9.85 **	** 0.385±0.004 **
Normal	181.46±15.76	0.406±0.005	193.97±19.07	0.445±0.011	** 144.45±11.57 **	** 0.330±0.007 **

To further examine possible memorization and sample diversity, an SSIM-based validation was conducted for the FastGAN-generated SDP images, as shown in Table 8. For each generated image, the nearest-neighbor SSIM with the 20 real support images of the same class was calculated. The overall nearest-neighbor SSIM was 0.508±0.052, and no exact duplicate was found between generated and real support images. In addition, the pairwise SSIM among generated images was 0.608±0.115. These results indicate that the generated samples were not direct copies of the limited support images and retained intra-class diversity.

**Table 8:** SSIM-based memorization and diversity validation of FastGAN-generated SDP images.

Sample label	Real support	Generated	Exact duplicates	Nearest real-support SSIM	Generated pairwise SSIM
N	20	100	0	0.440±0.029	0.551±0.095
IR_0	20	100	0	0.482±0.037	0.541±0.057
OR_0	20	100	0	0.482±0.024	0.566±0.047
BO_0	20	100	0	0.495±0.033	0.588±0.061
IR_1	20	100	0	0.536±0.026	0.645±0.048
OR_1	20	100	0	0.528±0.032	0.580±0.055
BO_1	20	100	0	0.594±0.010	0.852±0.032
IR_2	20	100	0	0.547±0.020	0.692±0.038
OR_2	20	100	0	0.513±0.041	0.551±0.059
BO_2	20	100	0	0.464±0.028	0.513±0.107
Overall	200	1000	0	0.508±0.052	0.608±0.115

Nearest real-support SSIM was calculated between each generated image and the 20 real support images from the same class. Generated pairwise SSIM was calculated among generated images within each class.

#### Swin-T model experiment on few-shot samples.

In practical engineering applications, bearings operate under complex working conditions, and faults occur unexpectedly. Before the acquisition system is shut down, it records a large amount of normal operation data, while fault samples are relatively rare. For each class, 20 real support images were used for classifier training, and 20, 50, or 100 FastGAN-generated auxiliary images were added to simulate different levels of data augmentation. The same held-out real validation and test sets described above were used for all models. After 300 training epochs, the test accuracy of each model under the three augmentation settings was obtained, as shown in Fig 11. The comparison included a convolutional neural network (CNN), AlexNet, VGG-16, and Swin-T, and the corresponding comparison results are listed in Table 9.

**Table 9:** Test accuracy of different models across varying small-sample proportions.

Auxiliary samples per class	Diagnostic Model Accuracy			
	CNN	AlexNet	VGG-16	Swin-T
20	0.621	0.661	0.812	**0.901**
50	0.681	0.719	0.837	**0.928**
100	0.724	0.753	0.854	**0.944**

**Fig 11:**
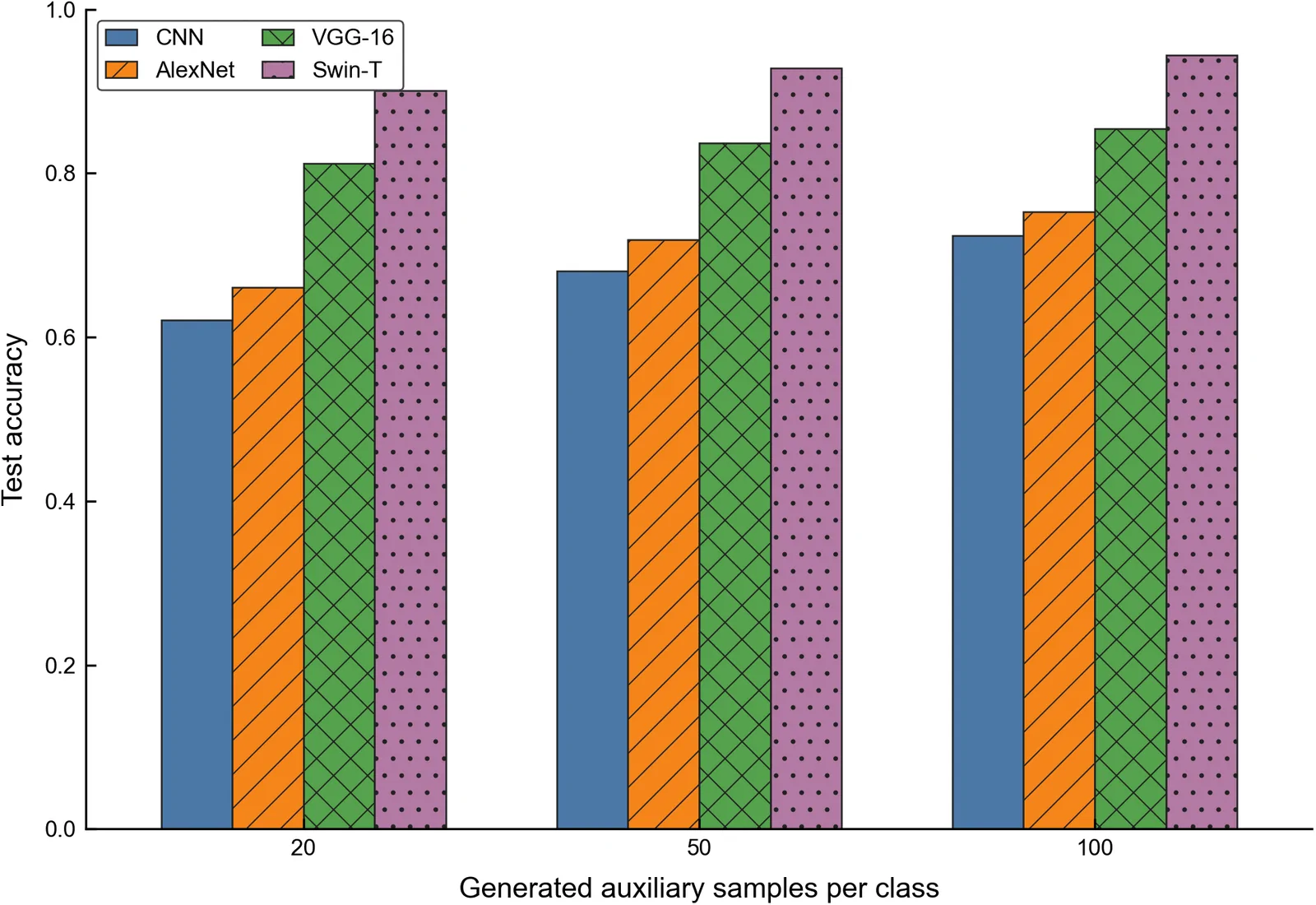
Diagnostic accuracy of various models with added auxiliary samples.

Diagnostic accuracy increases as more auxiliary samples are added. From 20 to 100 auxiliary samples per class, CNN, AlexNet, and VGG-16 increase by 10.3, 9.2, and 4.2 percentage points, respectively. The Swin-T model increases by 4.3 percentage points and reaches 94.4%, which is higher than the convolutional baselines. Fig 12 shows the Swin-T training-accuracy curves. All three settings converge rapidly, whereas the model with 100 auxiliary samples reaches a stable high-accuracy region earlier during training. These results support the use of FastGAN-generated SDP samples in limited-sample training, while also showing that the augmentation size matters.

**Fig 12:**
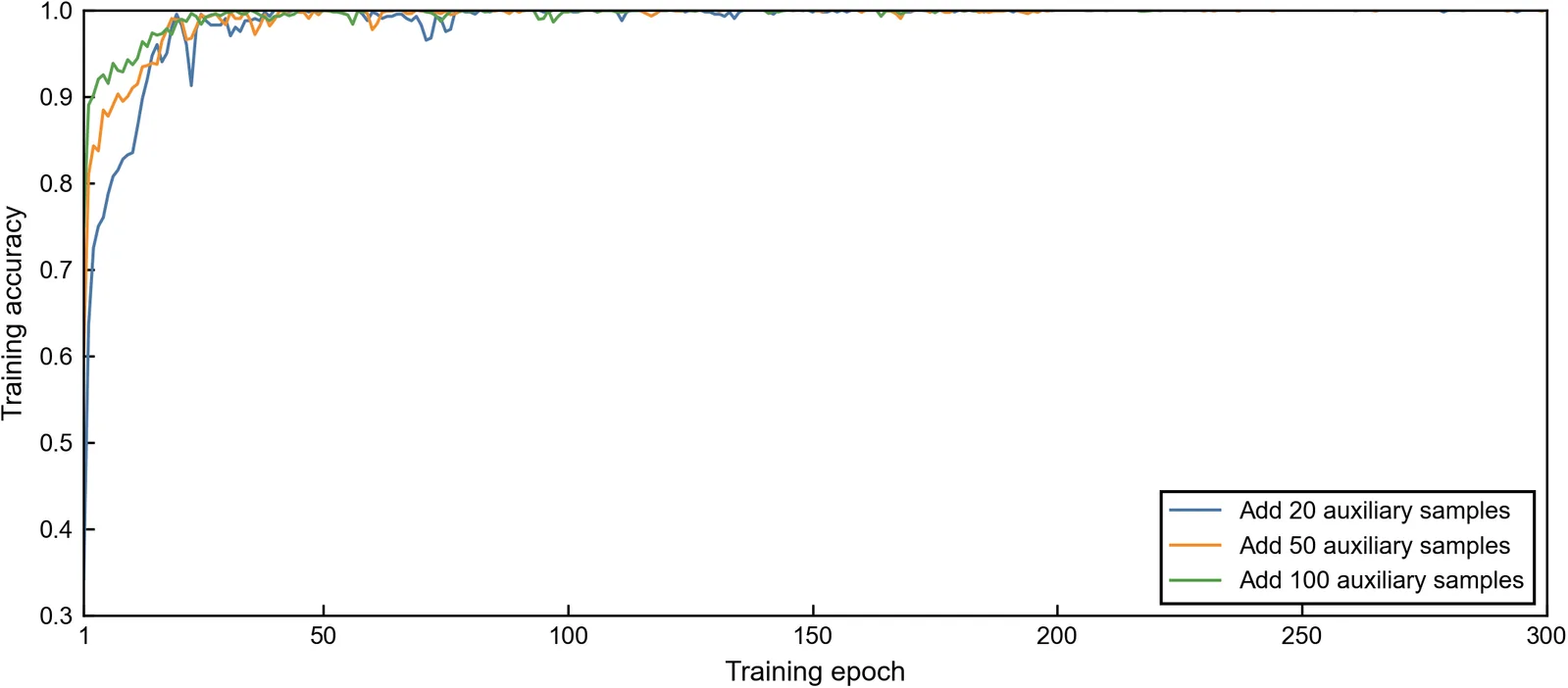
Training accuracy of the Swin-T model with different numbers of added auxiliary samples over 300 epochs.

To objectively assess the feature-extraction ability of the diagnostic model in the imbalanced few-shot CWRU setting with 100 auxiliary samples per class, t-distributed stochastic neighbor embedding (t-SNE) was applied to the features extracted by the Swin-T model. The plotted points represent held-out test samples rather than the generated auxiliary samples used for training. The category numbers 0–9 correspond to the fault types listed in Table 2. Feature visualization was performed after global model training using the final converged model parameters.

Fig 13 compares the original sample distribution with the Swin-T feature distribution. In Fig 13(a), samples from several fault classes overlap, and the class boundaries are unclear. In Fig 13(b), samples from the same fault category form more compact clusters, and most categories are separated more clearly in the t-SNE space. Some neighbouring categories remain relatively close, indicating residual feature similarity between fault modes. Overall, the visualization provides qualitative evidence that the Swin-T classifier improved feature separability in the imbalanced few-shot CWRU setting.

**Fig 13:**
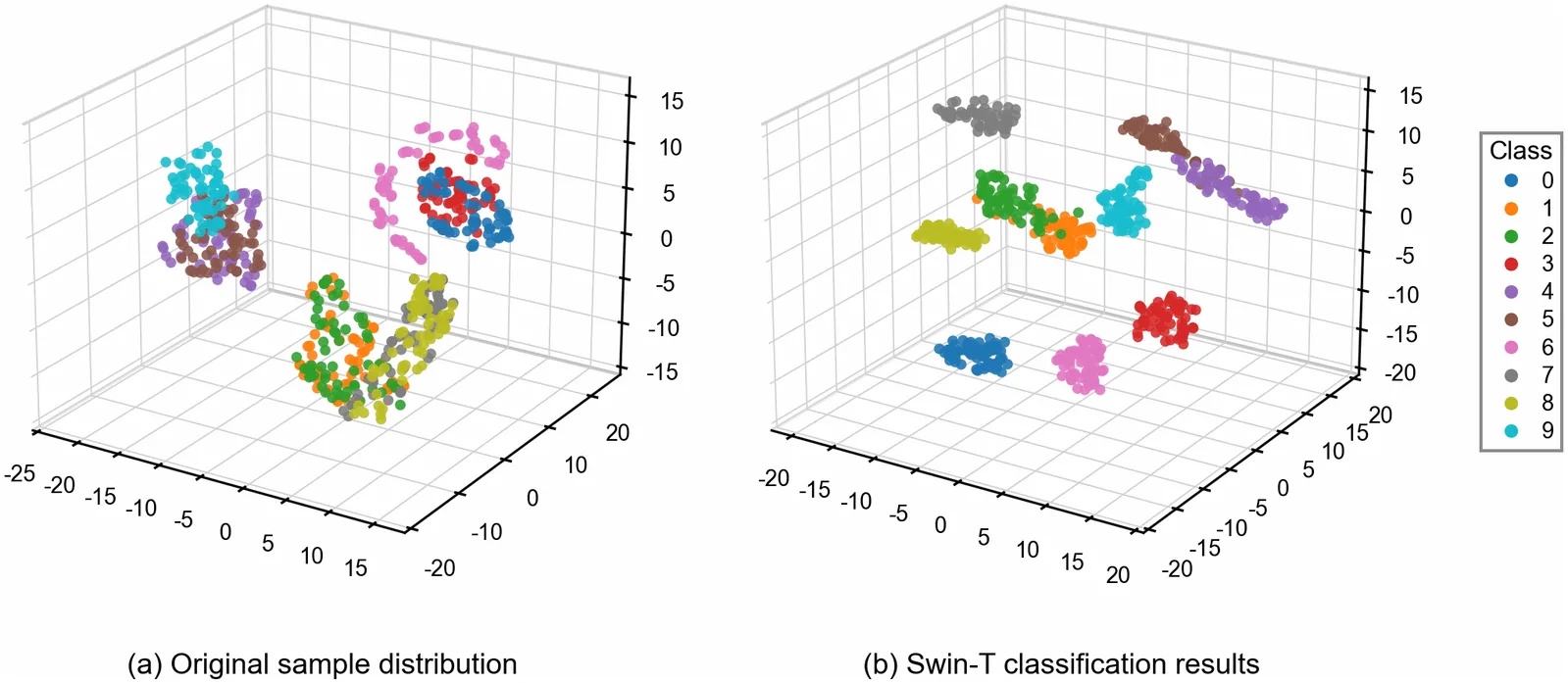
t-SNE visualization analysis of the Swin-T model.

### Ablation experiments

Ablation experiments were conducted to estimate how each module affects few-shot diagnosis performance. Because the generated-image comparison among FastGAN, DCGAN, and WGAN has already been reported, and because Swin-T is used as the fixed classifier in this part, this section focuses on VMD decomposition, RIME-based parameter optimization, and FastGAN-based data augmentation. The ablation settings are listed in Table 10. All models were trained five times with the same training parameters.

**Table 10:** Comparison results of different models.

Model	Model structure	Accuracy/%	Recall/%	F1-score/%
M1	SDP-Swin-T	84.73±1.86	84.31±1.93	84.09±2.15
M2	VMD-SDP-Swin-T	87.92±1.62	87.58±1.68	87.36±1.82
M3	RIME-VMD-SDP-Swin-T	90.61±1.35	90.28±1.42	90.13±1.55
M4	VMD-SDP-FastGAN- Swin-T	92.80±1.04	92.47±1.12	92.35±1.26
M5	RIME-VMD-SDP- FastGAN-Swin-T	**94.40 ± 0.78**	**94.12 ± 0.84**	**94.28 ± 0.91**

To further verify the contribution of each module to CWRU few-shot learning, inter-module ablation experiments were conducted, and each experiment was repeated five times. The results are reported as the mean ± standard deviation in Table 10. The results show that introducing VMD improves model M2 over the baseline M1 by 3.19 percentage points, indicating that signal decomposition helps the classifier use fault-related components. After RIME-based VMD parameter optimization is added, model M3 further increases the diagnostic accuracy by 2.69 percentage points. Subsequently, model M4 uses 100 FastGAN-generated auxiliary samples per class and improves the accuracy to 92.80%. The complete model M5 reaches an average diagnostic accuracy of 94.40% and an F1-score of 94.28%. These results support the combined use of signal enhancement, parameter optimization, and sample augmentation in the CWRU limited-sample setting, while the cross-condition contribution is examined separately on XJTU-SY.

The computational overhead of the multi-stage framework is summarized in Table 11. Among the four stages, Swin-T training and evaluation required the longest runtime (569.02±4.69 s), followed by FastGAN training and evaluation (452.67±2.73 s). RVMD required 167.0±5.0 s, whereas SDP mapping showed the lowest time cost (35.0±10.0 s). RVMD and SDP are non-trainable signal-processing stages, so neural-network parameter numbers and neural-network FLOPs are not reported for these two stages. These results indicate that the main computational burden comes from sample generation and classifier training, whereas RVMD and SDP mainly contribute preprocessing overhead.

**Table 11:** Computational cost of the main stages in the proposed framework.

Metric	RVMD	SDP	FastGAN	Swin-T
Runtime	167.0±5.0 s	35.0±10.0 s	452.67±2.73 s	569.02±4.69 s
Parameter numbers	–	–	46.185 M	27.522 M
FLOPs	–	–	2.0G	4.508G

Runtime is mean ± standard deviation over five independent runs. RVMD runtime includes RIME-based parameter optimization and reconstruction, and SDP runtime refers to image mapping. RVMD and SDP are non-trainable signal-processing stages, so trainable parameter numbers and neural-network FLOPs are not reported for these two stages. FastGAN and Swin-T report trainable neural parameters and neural-network FLOPs. CMMD acts only as a training loss and adds no trainable parameters or inference layers.

### Case study 2

After RVMD denoising, the XJTU-SY signals listed in Table 3 were converted into the SDP images shown in Fig 14. The images differ in petal morphology, color saturation, and scatter distribution across operating conditions. In the following experiments, OR_1, CF_1, and IR_1 were used as source-domain classes, and OR_2, CF_2, and IR_2 were used as the corresponding target-domain classes under different speeds and loads. This setting tests whether a classifier can adapt when the fault type is shared but the operating condition changes.

**Fig 14:**
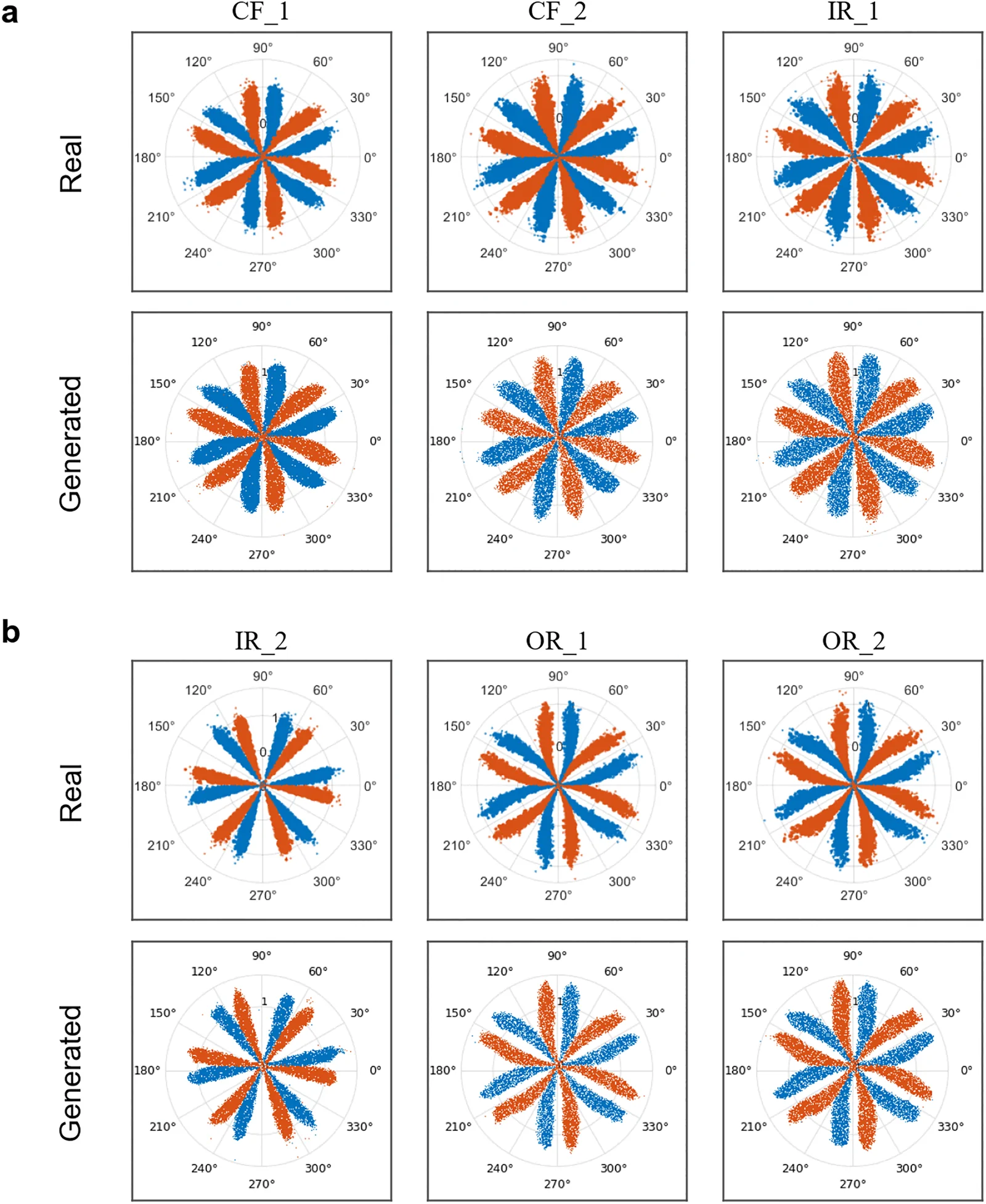
SDP Images of 6 Bearing States.

#### Source-only cross-condition generalization experiment.

To simulate an engineering scenario in which only a small number of samples from existing working conditions are available while samples from new working conditions are completely unknown, this experiment was conducted under a source-only setting. Specifically, 20 SDP images were randomly selected from each class as the source-domain and target-domain samples for the Swin-T model and the few-shot learning methods, Model-Agnostic Meta-Learning (MAML) and Prototypical Network (ProtoNet). The samples from the two domains were selected without overlap, and the target-domain samples were not involved in model training. All three methods were trained only on the source-domain samples and then directly evaluated on the target domain. Fig 15 presents the source-only Swin-T visualization used to examine the difficulty of direct cross-condition transfer.

**Fig 15:**
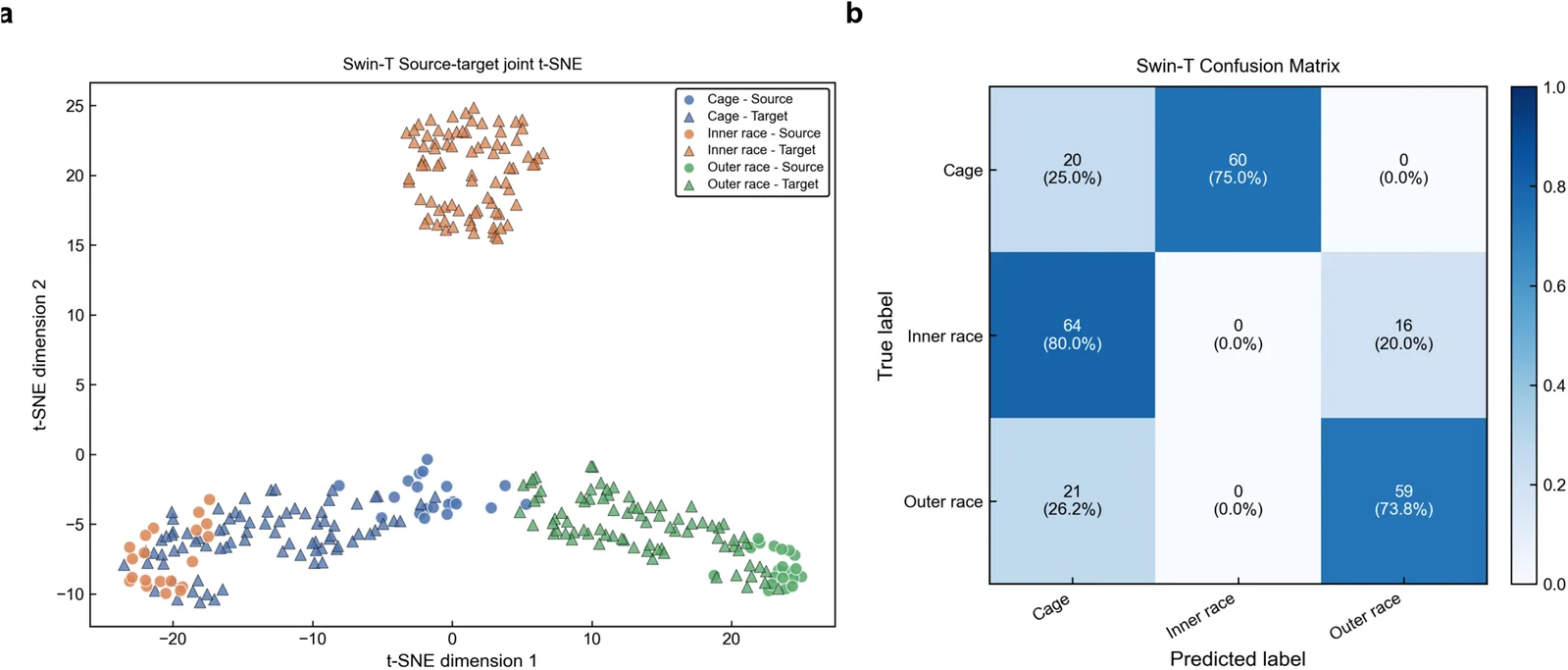
Source-only Swin-T diagnostic results under the cross-condition setting.

Fig 15 shows that direct cross-condition diagnosis using only the source-domain-trained Swin-T model was not ideal. The confusion matrix contains a large number of misclassified predictions, and the model achieved only 35.33% accuracy on the target domain. The source-target joint t-SNE visualization further shows that, although several source-domain samples formed local clusters, the target-domain samples were not well aligned with the corresponding source-domain fault categories. Therefore, relying only on the Swin-T model without target-domain adaptation is insufficient for this cross-condition diagnosis task. In the subsequent target-domain few-shot adaptation experiments, labelled target support samples, FastGAN-based target-domain augmentation, and CMMD-based class-wise alignment were introduced to improve the target-query diagnostic performance.

#### Target-domain few-shot cross-condition adaptation experiment.

The next experiment evaluated all methods under target-domain few-shot adaptation settings. The compared baselines included MAML, ProtoNet, dual prototypical contrastive network (DPCN), and GCDAN. These methods were selected as representative baselines rather than as an exhaustive state-of-the-art benchmark. MAML and ProtoNet represent classical few-shot learning strategies, DPCN represents a prototypical contrastive few-shot diagnostic model, and GCDAN represents a graph-based adversarial cross-domain diagnostic model. In addition, MMD and CORAL were used in the ablation study as common distribution-alignment baselines. Therefore, the comparison focuses on representative few-shot and cross-domain diagnosis settings under the same data split and evaluation protocol. For clearer distinction from the number of real labelled target-support samples, the two target-augmentation settings are denoted as G20 and G50. G20 and G50 indicate that 20 and 50 FastGAN-generated auxiliary target-domain samples were added per class, respectively. In both settings, the real labelled target-support set was fixed at 20 samples per class. Before FastGAN training and downstream adaptation, the real target-domain SDP images were partitioned into non-overlapping target-support and target-query sets, with no image shared between the two sets. To avoid the bias caused by different test sets, the same target-query samples were used for the G20 and G50 settings, and the G20 generated support set was selected as a subset of the corresponding G50 generated support set. In this study, target-support denotes the labelled real target-domain SDP images used to train FastGAN, whereas target-generated denotes the FastGAN-generated target-domain auxiliary samples used in the downstream adaptation stage. Target-query denotes the held-out real target-domain samples used only for final evaluation.

For each matched split, FastGAN was trained once using the same 20 real target-support SDP images per class. The trained generator was then used to produce auxiliary target-domain samples. Specifically, G20 used 20 generated auxiliary samples per class, whereas G50 used 50 generated auxiliary samples per class. Therefore, the principal difference between G20 and G50 was the number of generated target-domain auxiliary samples used for downstream classifier training and CMMD-based class-wise alignment.

The target-query set was excluded from FastGAN training, synthetic-sample selection, classifier training, CMMD optimization, hyperparameter adjustment, early stopping, and model selection, and was used only for final evaluation. All methods used identical source-domain samples, target-query samples, and evaluation procedures under each matched run. The experiments were repeated over five matched runs, and the results are reported as mean ± standard deviation. Paired statistical tests were used because the compared methods were evaluated on the same matched splits. The detailed data allocation is shown in Table 12.

**Table 12:** Sample allocation settings for target-domain few-shot cross-condition adaptation.

Category	Method	Source-real	Target-support	Target-generated	Target-query
		class	class	class	class
G20	MAML	100	20	0	80
	ProtoNet				
	DPCN				
	FastGAN+GCDAN			20	
	Proposed method				
G50	FastGAN+GCDAN	100	20	50	80
	Proposed method				

Fig 16 shows the confusion matrices and source-target joint t-SNE feature distributions of different models under the G20 setting. MAML and ProtoNet still produced obvious cross-class misclassifications, especially for Inner race and Cage samples, with diagnostic accuracies of 83.75% and 87.08%, respectively. This experiment further compared the proposed method with two existing few-shot cross-domain diagnostic models, DPCN and GCDAN. Under the same G20 target-augmentation setting and experimental configuration, GCDAN achieved 89.58% accuracy, which was lower than the 92.08% accuracy obtained by DPCN. The proposed method achieved 92.92%, the highest value among the evaluated methods in this setting. Its remaining errors were mainly concentrated in the Cage class, where several Cage samples were predicted as Inner race samples. The t-SNE feature distributions provide a consistent explanation for these results. The source-domain and target-domain features of MAML and ProtoNet showed elongated distributions or partial mixing, indicating insufficient class-wise alignment. Compared with DPCN and GCDAN, the proposed method formed more compact class-specific feature regions and clearer source-target correspondence. These results indicate that the generated auxiliary samples and CMMD-based class-wise alignment improved the target-domain feature representation. The G50 experiment further examined whether this trend remained when more generated auxiliary samples were introduced, and the results are shown in Fig 17.

**Fig 16:**
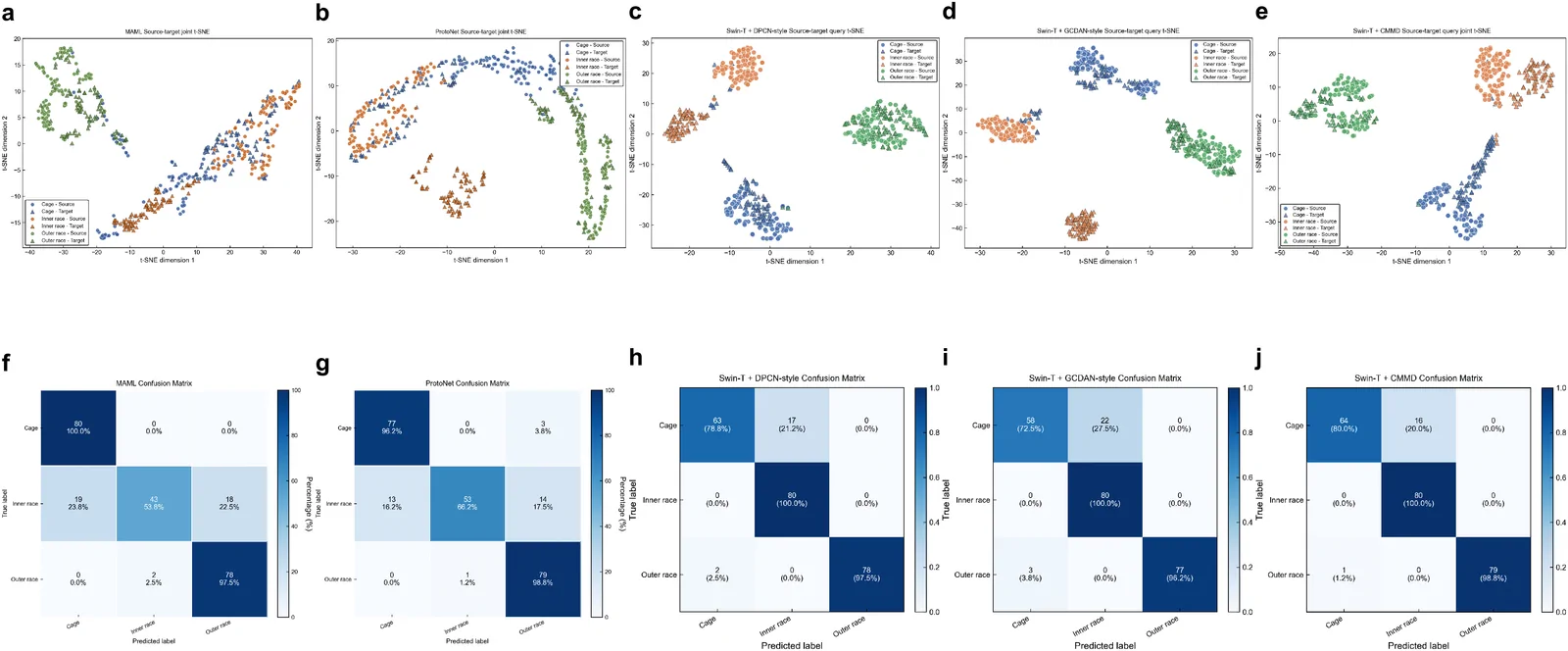
Confusion matrices and source-target joint t-SNE visualizations under the G20 target-augmentation setting.

**Fig 17:**
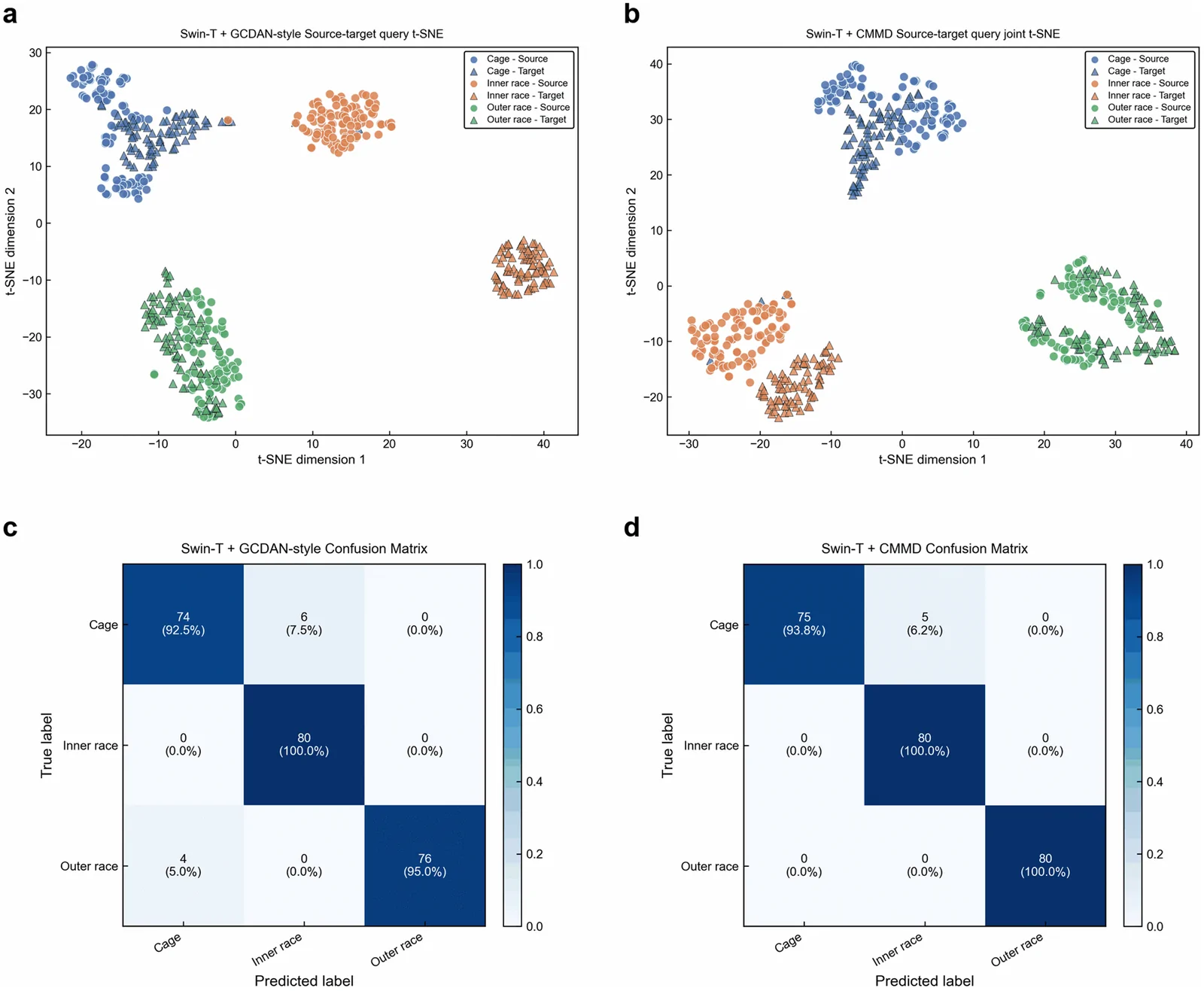
Confusion matrices and source-target joint t-SNE visualizations under the G50 target-augmentation setting.

Fig 17 shows the confusion matrices and source-target joint t-SNE feature distributions under the G50 setting. When the number of generated target-domain auxiliary samples was increased, the diagnostic performance of the generation-based models improved. Under the same target-augmentation setting, the GCDAN-based model achieved an accuracy of 95.83%, whereas the proposed method achieved 97.92%. The t-SNE visualizations further show that, compared with the G20 setting, the G50 setting produced more compact class clusters and clearer source-target correspondence. Among the evaluated methods, the proposed method showed clearer class-wise feature separation. These results indicate that increasing the number of generated auxiliary samples from 20 to 50 per class reduced target-query classification errors and improved feature separability. The averaged quantitative results over five matched runs are reported in Table 13.

**Table 13:** Quantitative comparison under target-domain few-shot cross-condition adaptation on the XJTU-SY dataset.

Method	Setting	Accuracy (%)	Recall (%)	Macro-F1 (%)
MAML	G20	80.33±13.96	80.33±13.96	75.51±19.56
ProtoNet	G20	88.83±4.81	88.83±4.81	88.40±5.09
Swin-T + DPCN	G20	93.08±2.27	93.08±2.27	93.04±2.31
FastGAN + GCDAN	G20	92.67±1.81	92.67±1.81	92.60±1.86
Proposed method	G20	93.33±1.33	93.33±1.33	93.20±1.40
FastGAN + GCDAN	G50	97.25±1.09	97.25±1.09	97.25±1.09
Proposed method	G50	97.58±0.68	97.58±0.68	96.58±0.69

Runtime is mean ± standard deviation over five independent runs. RVMD runtime includes RIME-based parameter optimization and reconstruction, and SDP runtime refers to image mapping. RVMD and SDP are non-trainable signal-processing stages, so trainable parameter numbers and neural-network FLOPs are not reported for these two stages. FastGAN and Swin-T report trainable neural parameters and neural-network FLOPs. CMMD acts only as a training loss and adds no trainable parameters or inference layers.

To ensure a fair comparison, each model was evaluated over five independent runs with different random seeds. Because MAML, ProtoNet, and Swin-T + DPCN do not use generated target-domain auxiliary samples, their results are reported using the G20 reference split. Among the evaluated baselines under the same data split and evaluation protocol, the proposed method achieved the highest diagnostic accuracy in both target-augmentation settings. In the G20 setting, the proposed method obtained an accuracy of 93.33±1.33%, which was higher than MAML, ProtoNet, Swin-T + DPCN, and FastGAN + GCDAN. In the G50 setting, the proposed method further achieved 97.58±0.68% accuracy, which was slightly higher than FastGAN + GCDAN under the same target-augmentation setting. In addition, the proposed method showed a lower standard deviation than FastGAN + GCDAN in diagnostic accuracy, indicating more stable performance across repeated runs. Overall, these results indicate that combining FastGAN-based target-domain augmentation with CMMD-based class-wise alignment improves target-query diagnosis in the evaluated cross-condition few-shot setting.

Statistical significance was evaluated using analysis of variance (ANOVA), Friedman tests, and false discovery rate (FDR)-corrected paired comparisons.

To distinguish the effects of FastGAN-based augmentation and feature alignment, we conducted the ablation experiments in Table 14 using five repeated runs with different random seeds. In the G20 setting, FastGAN and CMMD improved the Swin-T baseline accuracy by 0.45 and 1.25 percentage points, respectively. Their combination achieved the best accuracy of 93.33% among the evaluated variants and was higher than the global MMD and CORAL baselines. FDR-corrected paired tests showed significant improvements over Swin-T, Swin-T+FastGAN, Swin-T+global MMD, and Swin-T+CORAL for both accuracy and Macro-F1. In the G50 setting, only FastGAN-based variants were compared. The proposed method achieved the highest accuracy and Macro-F1 among these variants, reaching 97.58±0.68% and 97.45±0.70%, respectively. ANOVA also indicated significant overall differences in both comparison settings.

**Table 14:** Ablation and domain-alignment results on the XJTU-SY dataset.

Method	G20 Acc. (%)	G20 F1 (%)	G50 Acc. (%)	G50 F1 (%)
Swin-T	91.80±2.40	91.65±2.55	—	—
Swin-T + FastGAN	92.25 ± 2.10	92.05 ± 2.25	96.85 ± 0.95	96.60 ± 1.05
Swin-T + global MMD	92.40±1.95	92.20±2.05	—	—
Swin-T + FastGAN + global MMD	92.85±1.70	92.65±1.80	97.20±0.80	97.00±0.85
Swin-T + CORAL	92.55±1.85	92.35±1.95	—	—
Swin-T + FastGAN + CORAL	92.95±1.60	92.75±1.70	97.25±0.75	97.05±0.80
Swin-T + CMMD	93.05±1.45	92.90±1.55	—	—
Proposed method	** 93.33±1.30 **	** 93.20±1.40 **	** 97.58±0.68 **	** 97.45±0.70 **
ANOVA *p*	0.018	0.021	0.041	0.048
Friedman *p*	0.026	0.031	0.055	0.061

Dashes denote non-applicable settings for methods without FastGAN-generated auxiliary samples. Analysis of variance (ANOVA) and Friedman tests assess overall differences; G50 tests include only FastGAN-based variants. False discovery rate (FDR)-corrected paired tests were used for post hoc comparisons.

Fig 18 links diagnostic accuracy with feature-space alignment. Among the evaluated variants, the proposed method gave the highest accuracy in both target-augmentation settings. Its accuracy increased from 93.33% with 20 generated samples per class to 97.58% with 50 generated samples per class. The centroid-distance analysis further showed that FastGAN and CMMD produced complementary effects. FastGAN alone moderately reduced the source-target distance, CMMD alone gave a larger reduction, and their combination produced the smallest distances across all three fault categories.

**Fig 18:**
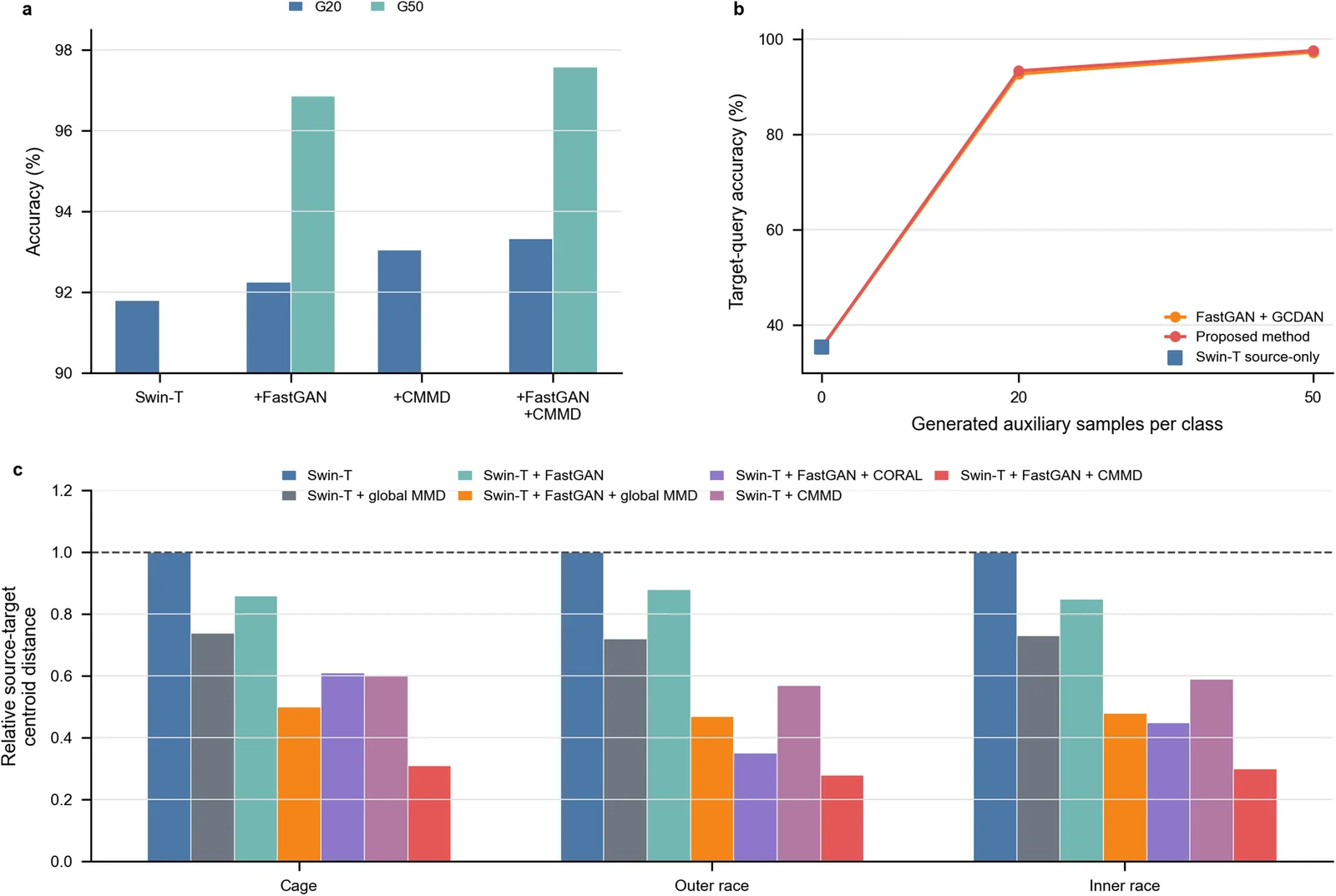
Evidence for target-domain augmentation and class-wise alignment on the XJTU-SY dataset. (a) Ablation accuracy under G20 and G50 target-augmentation settings. (b) Target-query accuracy versus the number of generated auxiliary samples per class. (c) Normalized class-wise source-target centroid distance in the G20 setting. Centroid distances were computed using only real source samples and held-out real target-query samples, excluding FastGAN-generated samples.

## Discussion

The experiments point to a practical limitation of direct source-only diagnosis. The problem is not simply that the target condition has few samples; source and target features also occupy different class-wise distributions. In the XJTU-SY source-only setting, the Swin-T model showed poor direct cross-condition performance. Although MAML and ProtoNet improved the target-domain results after few-shot adaptation, their feature distributions still showed incomplete source-target alignment. This observation defines the main scope of the proposed method: it is intended for situations where a small labelled target support set can be collected before deployment.

The framework addresses this setting in two steps. FastGAN expands the target-domain representation in the SDP image space, and CMMD aligns source and target features within each fault category. The centroid-distance comparison separates these effects while excluding generated samples from the distance calculation. This design tests whether augmentation changes the subsequent alignment effect without using the target query set during training.

RIME-VMD should be interpreted as a signal enhancement step, not as the sole reason for the diagnostic improvement. The CWRU ablation results show that denoising and adaptive parameter optimization improve the SDP representation, whereas the cross-condition gain mainly depends on how the target condition is used during adaptation. The feature-distance analysis on XJTU-SY gives the same message from another angle. The main observed change was the reduction of class-wise source-target mismatch after target-domain augmentation and CMMD were used together. The strict separation between target support and target query also helps ensure that this gain reflects adaptation to the target condition rather than direct exposure to the final test samples.

The study has several limitations. FastGAN-based augmentation depends on labelled target support samples, so its reliability may decrease when the support set is extremely small or contains label noise. Although representative few-shot and cross-domain baselines were included, the comparison is not intended to be an exhaustive state-of-the-art benchmark. Recent digital-twin-assisted, graph-contrastive, self-supervised, and knowledge-distillation-based diagnostic frameworks may have different data requirements and training protocols. The XJTU-SY validation also focuses on a specific source-target operating-condition transfer, and broader transfer directions are needed to characterize generalization more fully. Field data with less controlled noise, unseen fault severities, and mixed operating conditions are also needed before the method can be treated as deployment-ready. Future work will further evaluate the proposed framework against broader recent cross-domain and few-shot fault diagnosis methods under unified source-target splits and implementation settings.

## Conclusions

This study presented a few-shot cross-condition bearing fault diagnosis framework based on signal-enhanced SDP representation, target-domain sample augmentation, and class-wise feature alignment. RIME-optimized VMD enhanced the fault-sensitive vibration components, and SDP mapping converted the reconstructed signals into polar-coordinate images. FastGAN generated auxiliary samples from the limited target support set, while CMMD aligned source and target features within the same fault category.

Experiments on the CWRU and XJTU-SY datasets indicated that the framework improved diagnostic performance under the evaluated limited-sample and cross-condition settings. In the XJTU-SY target-domain adaptation task, the proposed method achieved 93.33% and 97.58% accuracy in the G20 and G50 target-augmentation settings, respectively. The ablation and feature-distance results further explain the role of the two main adaptation components: FastGAN enriched the target-domain class representation, whereas CMMD reduced the remaining class-wise source-target mismatch after augmentation.

The framework is intended for bearing fault diagnosis scenarios where source-condition data are available and a small labelled target support set can be collected. Future work will focus on industrial field validation, broader comparisons with recent domain-adaptation baselines, and robustness analysis under more complex operating conditions.

## Abbreviations

ANOVA: Analysis of variance
IMF: Intrinsic mode function
BO: Ball fault
IR: Inner race fault
CC: Correlation coefficient
MAML: Model-agnostic meta-learning
CE: Cross-entropy
MMD: Maximum mean discrepancy
CMMD: Class-wise maximum mean discrepancy
N: Normal state
CNN: Convolutional neural network
NCC: Normalized cross-correlation coefficient
CORAL: Correlation alignment
OR: Outer race fault
CSSCL: Cross-sensor self-contrastive learning
ProtoNet: Prototypical network
CWRU: Case Western Reserve University
PSO: Particle swarm optimization
DCGAN: Deep convolutional generative adversarial network
RIME: Rime optimization algorithm
DCGKDN: Dual-correlation graph knowledge-distillation network
RMSE: Root mean square error
DPCN: Dual prototypical contrastive network
RVMD: RIME-optimized variational mode decomposition
FastGAN: Fast generative adversarial network
SDP: Symmetric dot pattern
FDR: False discovery rate
SLE: Skip-layer channel excitation
FID: Fréchet inception distance
SNR: Signal-to-noise ratio
GA: Genetic algorithm
Swin-T: Swin Transformer
GAN: Generative adversarial network
VMD: Variational mode decomposition
GCDA: Graph contrastive domain adaptation
WGAN: Wasserstein generative adversarial network
GCDAN: Graph convolutional domain adversarial network
XJTU-SY: Xi’an Jiaotong University–Shenyang Blower Works
